# Hemodynamic impact of blood viscosity in intracranial atherosclerotic arteries with varying stenosis severity: A non-newtonian computational fluid dynamics patient specific study

**DOI:** 10.1371/journal.pone.0342713

**Published:** 2026-05-28

**Authors:** Lei Zhengyao, Muhammad Nor Ikmal Muhamad Nazri, Adi Azriff Basri, Masaaki Tamagawa, Kaito Sagara, Abdul Hanif Khan Yusof Khan, Eusni Rahayu Mohd Tohit

**Affiliations:** 1 Department of Aerospace Engineering, Faculty of Engineering, Universiti Putra Malaysia, Serdang, Selangor, Malaysia; 2 Department of Biological Functions Engineering, Kyushu Institute of Technology, Fukuoka, Japan; 3 Department of Medicine, Faculty of Medicine and Health Sciences, Universiti Putra Malaysia, Serdang, Selangor, Malaysia; 4 Department of Pathology, Faculty of Medicine and Health Sciences, Universiti Putra Malaysia, Serdang, Selangor, Malaysia; Universidade de Lisboa Instituto Superior Tecnico, PORTUGAL

## Abstract

Intracranial atherosclerotic stenosis (ICAS) is a major cause of ischemic stroke, yet geometric stenosis alone may not fully reflect the functional hemodynamic burden of a lesion. This study used computational fluid dynamics (CFD) with a shear-thinning non-Newtonian Carreau viscosity model to quantify the combined effects of stenosis severity and blood viscosity on intracranial hemodynamics. A middle cerebral artery (MCA) stenosis model with an original ~70% narrowing was reconstructed from computed tomography angiography, and additional idealized stenosis variants (30%, 50%, and 90%) were generated on the same anatomical background to enable controlled comparisons. Three viscosity states (below-normal, normal, and high) were simulated under transient, incompressible, laminar flow with rigid walls and identical boundary conditions. Velocity, pressure, and wall shear stress (WSS), oscillatory shear index (OSI) and time-averaged wall shear stress (TAWSS) were evaluated. The results show that flow behavior is governed by the combined influence of geometry and rheology, rather than by stenosis severity alone. Severe stenosis produced a dual pathological shear environment, characterized by elevated WSS within the stenotic region and disturbed low-shear flow downstream. In addition, TAWSS showed a non-monotonic response, increasing up to 70% stenosis and then decreasing at 90% stenosis. OSI also showed viscosity-dependent elevation under severe stenosis, with values ranging from approximately 0.38 to 0.48, indicating enhanced oscillatory and disturbed flow.These findings support integrating non-Newtonian hemorheology and hemodynamic metrics with geometric assessment to improve ICAS risk stratification and inform hemodynamics-guided intervention timing.

## 1. Introduction

Intracranial atherosclerotic stenosis Disease (ICAD) is one of the major causes of ischemic stroke, especially in the Asian population, with a proportion of 30–50% [1].In clinical practice, vascular stenosis rate is usually used as the core basis for disease severity and intervention decision, but a large number of studies have shown that only geometric stenosis degree is not enough to fully represent the real blood flow state in the diseased vessel: Different flow velocity distribution, pressure gradient and wall shear stress (WSS) patterns can affect endothelial function, inflammatory response and plaque evolution, thereby changing the risk and prognosis of stroke [2]. Therefore, hemodynamic assessment based on computational fluid dynamics (CFD) has gradually become an important means to understand the mechanism of ICAD and assist risk stratification. CFD technology provides a cost-effective and non-invasive method to study intravascular hemodynamics [3]. These simulations can display almost any parameter of interest to a medical practitioner.

Blood is a non-Newtonian fluid that contains platelets, white blood cells, red blood cells, and other components [4], however, researchers have considered blood as a Newtonian fluid. Nevertheless, in accordance with the blockage artery conditions, blood should be considered as a non-Newtonian nature [5]. Red blood cells may aggregate at low shear rates, exhibiting the characteristics of non – Newtonian fluids [6]. The Casson equation [11,12–15], power – law model [9], and Carreau – Yasuda model [10] are frequently employed in numerical studies of blood circulation dynamics.. Consequently, it is essential to select a model for the accurate evaluation of hemodynamics.

Although blood may behave approximately as a Newtonian fluid in high-shear regions such as the stenotic throat, this approximation may not remain valid across the entire flow domain. In post-stenotic, near-wall, and bifurcating regions, flow separation, recirculation, and locally reduced shear rates may occur, where shear-thinning effects become more relevant. This is particularly important when evaluating wall-based hemodynamic metrics such as WSS, TAWSS, and OSI, which are sensitive to local viscosity variation. Previous studies on intracranial arterial stenosis have shown that Newtonian and non-Newtonian models may produce similar pressure-related results in high-shear regions, whereas more pronounced differences arise in low-WSS and recirculating regions [11]. Therefore, a Carreau-type non-Newtonian model was adopted in the present study to better capture the spatial heterogeneity of blood rheology across stenotic, post-stenotic, and bifurcating regions.

In the majority of Computational Fluid Dynamics (CFD) studies, blood viscosity is typically set as a constant. However, in clinical practice, it cannot be ignored that diabetes will increase blood viscosity, leading to increased blood flow resistance, elevated capillary fragility, impaired platelet function, and vascular obstruction [5–8]. These changes will result in alterations in hemodynamics. Similarly, anemia reduces blood viscosity. Low – viscosity blood exerts less shear stress and pressure, enabling the heart to work in a compensatory manner [16]. Therefore, the viscosity effect also requires attention.

Few studies have systematically quantified the interaction between stenosis severity and inter-individual viscosity variation within the same geometric setting using a non-Newtonian model [17]. In the present study, the Carreau model, a widely used non-Newtonian hemorheological model, was adopted to investigate different stenosis severities (30%, 50%, 70%, and 90%) under below-normal, normal, and high viscosity conditions. Velocity distribution, pressure characteristics, and WSS variation were systematically compared across these combined scenarios to provide a more comprehensive hemodynamic interpretation of ICAD from a non-Newtonian perspective. It should be noted that the aim of this study was not to reproduce the full spectrum of patient-specific plaque remodeling and downstream vascular adaptation, but rather to perform a controlled parametric investigation of how stenosis severity and blood viscosity jointly influence intracranial hemodynamics within a consistent anatomical framework.

## 2. Materials and methods

### 2.1. 3D modelling of ICAD

**Fig 1:**
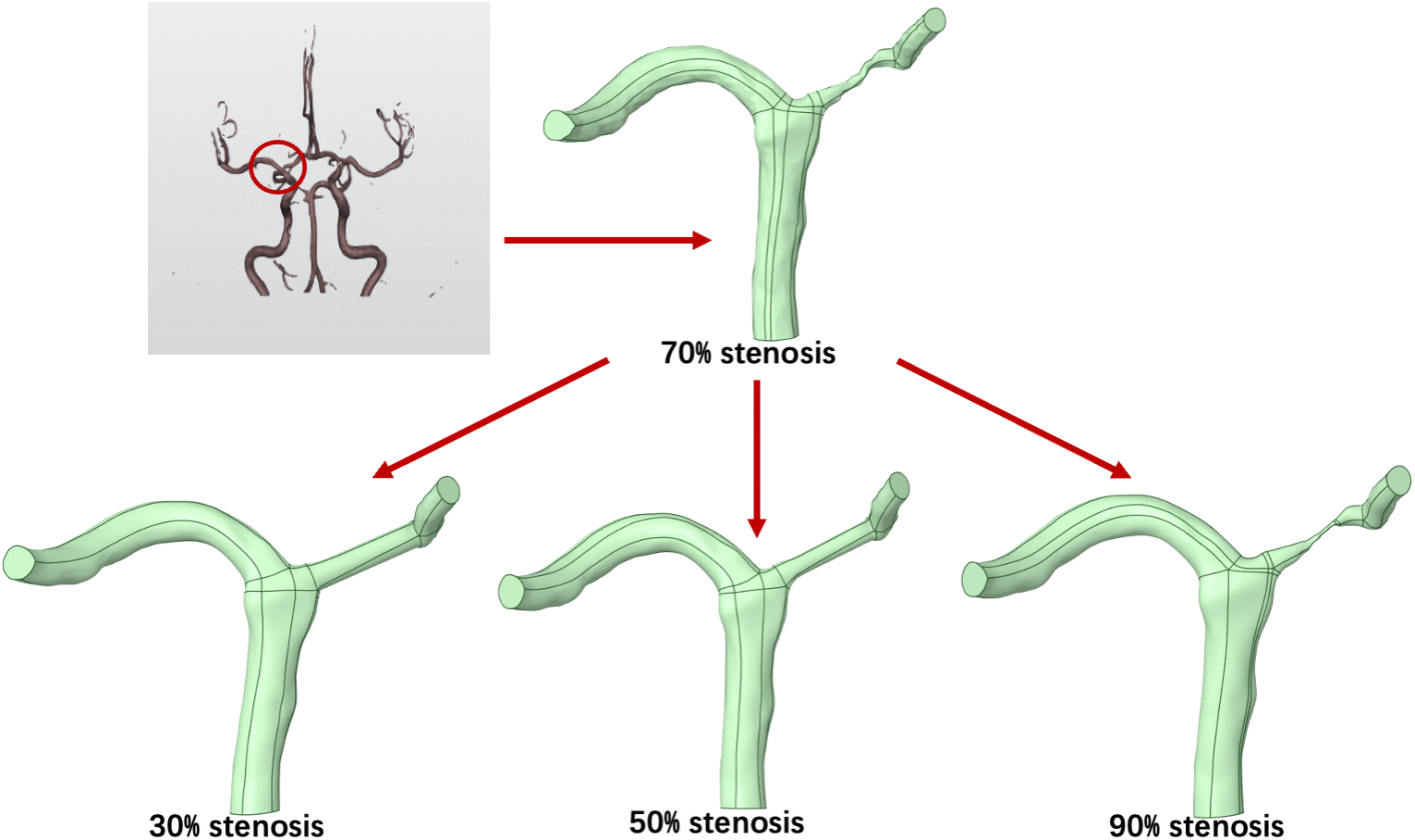
Reconstruction of the patient-derived intracranial arterial geometry from CTA data and generation of simplified stenosis variants (30%, 50%, and 90%) on the same anatomical background. The original bifurcating model exhibits approximately 70% stenosis, and the additional models were created by locally modifying stenosis severity for controlled comparison.

A three-dimensional intracranial arterial geometry was reconstructed from computed tomography (CT) images obtained at Sultan Abdul Aziz Shah Hospital (HSAAS). The target vessel was a middle cerebral artery (MCA) segment presenting an approximately 70% luminal stenosis in the original dataset, which served as the baseline model for subsequent analyses. To enable a controlled comparison of hemodynamic responses across stenosis severities under a consistent anatomical context, three additional geometric variants were generated from the baseline model to represent 30%, 50%, and 90% stenosis, respectively (Fig 1). It should be emphasized that the computational domain was based on a patient-derived bifurcating intracranial artery geometry rather than a purely straight-pipe model. The idealization in the present study was limited to the local modification of stenosis severity on the same anatomical background for controlled comparison.

**Fig 2:**
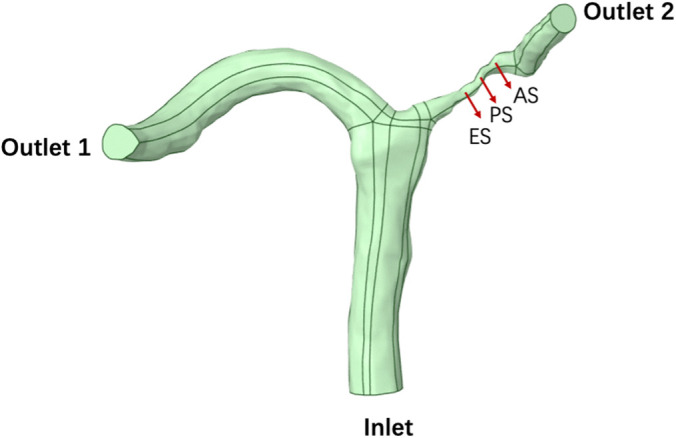
Three-dimensional reconstructed intracranial artery geometry showing the inlet, two outlets, and the characteristic cross-sections used for hemodynamic analysis. Three representative sections were defined along the stenotic branch: ES (early-stenotic section), PS (peak stenosis section), and AS (after-stenotic section). The same section definitions were applied consistently across all stenosis and viscosity cases.

Image segmentation and surface processing (including smoothing and artifact removal) were performed to obtain a watertight computational domain suitable for numerical simulation. All cases were simulated using ANSYS Fluent 2023 R2(ANSYS Inc., Canonsburg, PA, USA) with a non-Newtonian Carreau viscosity model. Identical boundary conditions and mesh resolution were applied across all stenosis configurations to ensure comparability of hemodynamic metrics.Three cross-sections were defined for post-processing: **ES** (early-stenosis), **PS** (stenotic throat at peak stenosis), and **AS** (after-stenosis)(Fig 2).

### 2.2. Mesh dependency/discretization

A mesh-independence study was conducted on the baseline 70% stenosis model, which represented the most hemodynamically critical case. The computational domain was discretized in ANSYS ICEM-CFD using an unstructured tetrahedral mesh, and the same meshing strategy was applied to all other stenosis models to ensure consistency and comparability. Hemodynamic simulations were performed in ANSYS Workbench/Fluent 2023. Numerical convergence was achieved when the scaled residuals fell below 1 × 10 ⁻ ⁵ and the monitored quantities showed no further appreciable change.

**Fig 3:**
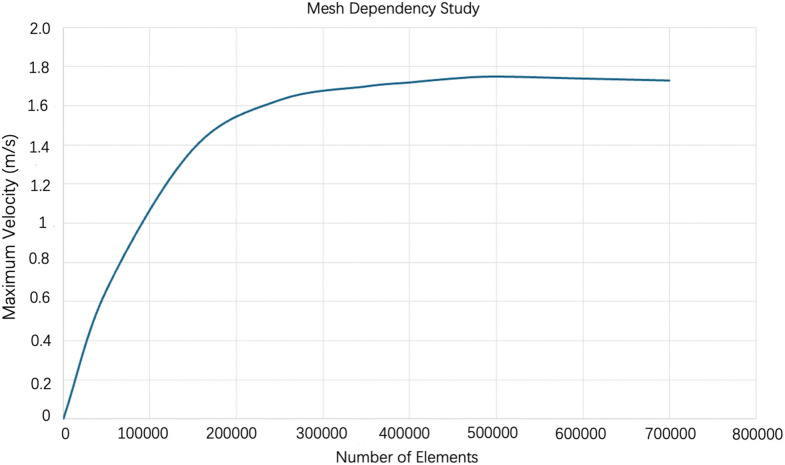
Mesh dependency analysis for the baseline 70% stenosis model, showing the variation of maximum velocity with mesh density.

The maximum velocity was first used to evaluate the global stability of the solution under mesh refinement. As the mesh density increased, the maximum velocity gradually approached a stable value, indicating that the overall flow prediction became insensitive to further refinement (Fig 3).

**Fig 4:**
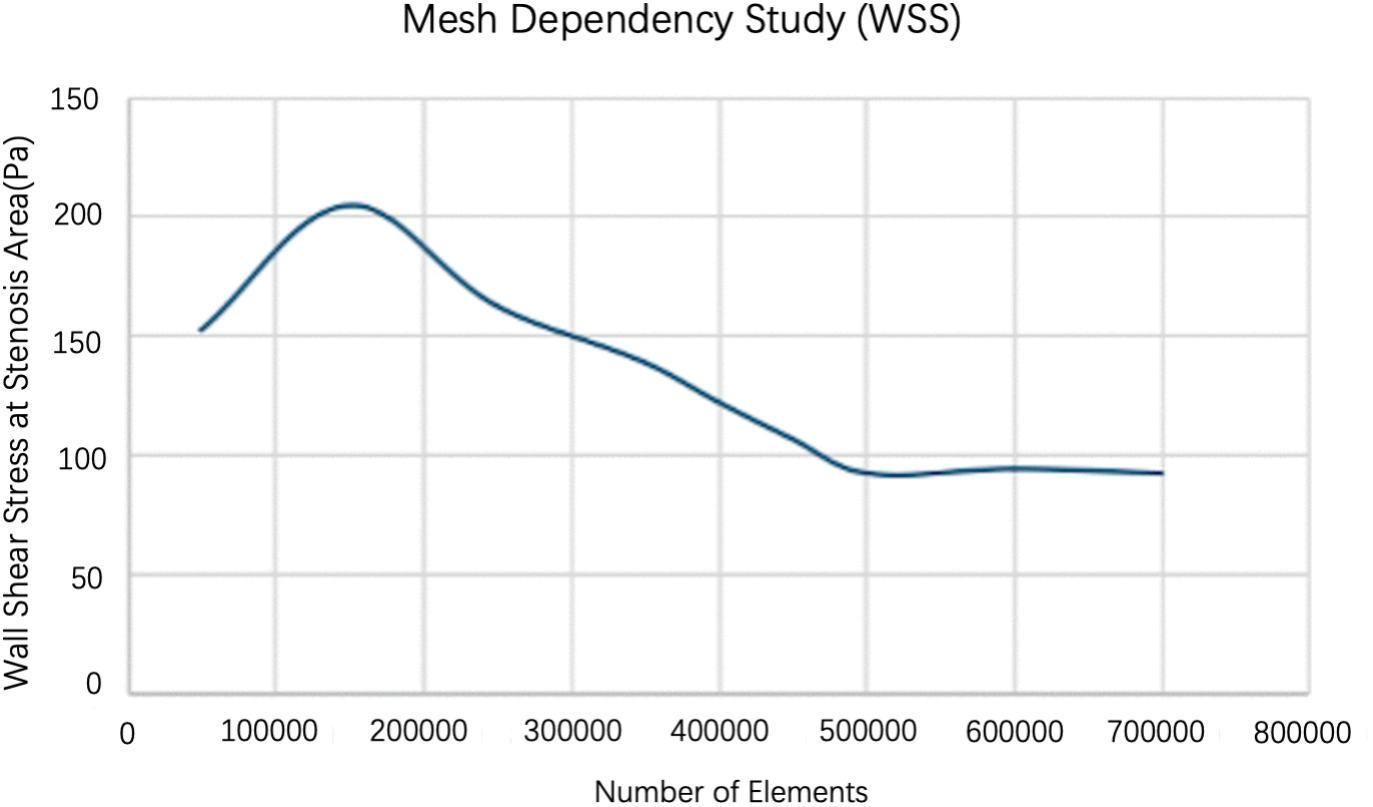
Mesh dependency analysis for the baseline 70% stenosis model, showing the variation of wall shear stress (WSS) with mesh density. The stabilization of WSS with mesh refinement indicates adequate near-wall resolution.

In addition, wall shear stress (WSS) at the stenotic region was used as a local indicator because of its higher sensitivity to near-wall velocity gradients (Fig 4). The WSS values became effectively stable when the mesh density exceeded approximately 5.0 × 10⁵ elements, suggesting that the near-wall resolution was sufficient. Therefore, the mesh with approximately 5.0 × 10⁵ elements was selected for all simulations as a compromise between numerical accuracy and computational cost.

### 2.3. Mathematical formulas

Blood was modeled as a three-dimensional, steady transient, laminar, and incompressible non-Newtonian fluid governed by the incompressible Navier–Stokes equations [18, 19]. The shear-thinning behavior of blood was represented using the Carreau model [20], where viscosity is a function of the shear rate $\dot{r}$ rather than a constant [21].


$$\begin{array}{c} \mu (\dot{r})=\mu \infty +(\mu 0-\mu \infty )[1+(\lambda \dot{r})2]2n-1 \end{array}$$

We used the Carreau model described by the above formula to study different blood conditions, including below-normal viscosity, normal viscosity, and high viscosity, with specific values shown in Table 1.where μ($\dot{r}$)is the apparent viscosity, μ0 and μ∞ are the zero- and infinite-shear viscosities, λ is the time constant, n is the power-law index, and$\dot{\;r}$ is the shear rate.

**Table 1:** Model parameters, boundary conditions, and numerical setup used in the simulations.

Category	Parameter	Value	reference
**Blood density**	ρ	1060 kg·m^-3^	[22]
**Rheology model**	Carreau model	–	
**low-viscosity**	Power-law index (n)	0.33	[6]
	Time constant(λ)	12.448s	[6]
	Zero-shear viscosity(μ0)	0.0178 Pa·s	[6]
	Infinite-shear viscosity(μ∞)	0.00257 Pa·s	[6]
**Normal-viscosity**	Power-law index (n)	0.3568	[6]
	Time constant(λ)	3.313s	[6]
	Zero-shear viscosity(μ0)	0.056 Pa·s	[6]
	Infinite-shear viscosity(μ∞)	0.0035 Pa·s	[6]
**High-viscosity**	Power-law index (n)	0.39	[6]
	Time constant(λ)	103.09s	[6]
	Zero-shear viscosity(μ0)	0.8592 Pa·s	[6]
	Infinite-shear viscosity(μ∞)	0.00802 Pa·s	[6]
**Boundary condition**	Inlet (velocity-inlet)	Pulsatile velocity waveform implemented via UDF	[23]
	Outlet1&2 (pressure-outlet)	Flow-dependent resistance-based outlet condition implemented via UDF	[23]
**Numerical setup**	Solver type	transient	
	Flow model	Laminar	
	Residual threshold	1 × 10 ^− 5^	
**Transient setting**	Cardiac cycle	1s	
	Time-step size	0.002s	
	Number of time steps	500	

A common definition for the shear rate [24] is:


$$\begin{array}{c} \dot{r}=\sqrt{2D:D\;},D=\frac{1}{2}(\nabla u+{\left(\nabla u\right)}^{T}) \end{array}$$

To capture pulsatility-related hemodynamic behavior, transient simulations were additionally performed using a cardiac cycle of 1 s, discretized into 500 time steps(Δt=0.002s). The final cycle was used for post-processing to ensure periodic stability. The time-averaged wall shear stress (TAWSS) was computed as [25]


$$\begin{array}{c} TAWSS=\frac{1}{T}\int_{0}^{T}\left|Tw(t)\right|dt \end{array}$$

The oscillatory shear index (OSI) was computed as [26]:


$$\begin{array}{c} OSI=0.5\times \left(1-\frac{\sqrt{\overset{2}{\underset{x,avg}{T}}+\overset{2}{\underset{y,avg}{T}}+\overset{2}{\underset{z,avg}{T}}}}{Tmag,avg}\right) \end{array}$$

The flow regime in the present study was further characterized using the Reynolds number, defined as [27]:


$$\begin{array}{c} Re=\frac{\rho 𝐔D}{\mu } \end{array}$$

where ρ is the blood density, 𝐔 is the characteristic velocity, D is the characteristic vessel diameter, and μ is the apparent viscosity. According to classical internal flow theory, the flow regime can be classified as laminar for Reynolds numbers below 2300, transitional between 2300 and 4000, and turbulent above 4000 (White, 2011) [28]. Based on the representative flow conditions in the present study, the Reynolds number was approximately 748,which is well below the commonly used transition threshold of Re = 2300; therefore, the flow was reasonably treated as laminar in the present simulations. Based on the simulated velocity ranges and vessel dimensions, the Reynolds number in the present models remained within a range consistent with laminar flow conditions typically reported in intracranial arteries. Therefore, a laminar flow assumption was adopted in the present simulations [29].

### 2.4. Boundary condition

The Carreau parameters, densities, and boundary condition configurations involved in the simulations are summarized in Tables 1 and 2. For the revised simulations, physiologically informed boundary conditions were applied using user-defined functions (UDFs). For the fluid domain, velocity and pressure were defined at the inlet and outlet, respectively, based on a literature-derived pulsatile blood flow model reported by Basri et al. (2020) [23]. The inlet condition was prescribed as a velocity waveform, while the outlet pressures were no longer imposed as equal constants. Instead, outlet pressure was dynamically adjusted according to a flow-dependent resistance formulation, allowing a more realistic coupling between local hemodynamics and downstream vascular impedance. This approach improves the physiological realism of branch flow distribution compared with equal-pressure outlet conditions.

**Table 2:** Quantitative comparison of key hemodynamic parameters between the present simulations and the clinically grounded CFD study reported by Leng et al. (2014).

	Reference study	Present study	Relative difference (%)
Pressure ratio	0.790	0.876	10.95%
Velocity ratio	5.2	5.06	2.69%

The cardiac cycle duration was set to 1.0 s, and the time step was set to 0.002 s. A total of 500 cardiac cycles were simulated to minimize the influence of initialization and to ensure that the solution reached a periodic state. Time-averaged wall shear stress (TAWSS) and oscillatory shear index (OSI) were calculated from the final cardiac cycle. Unless otherwise stated, the reported transient hemodynamic results were extracted after periodic behavior had been established.

## 3. Results

**Fig 5:**
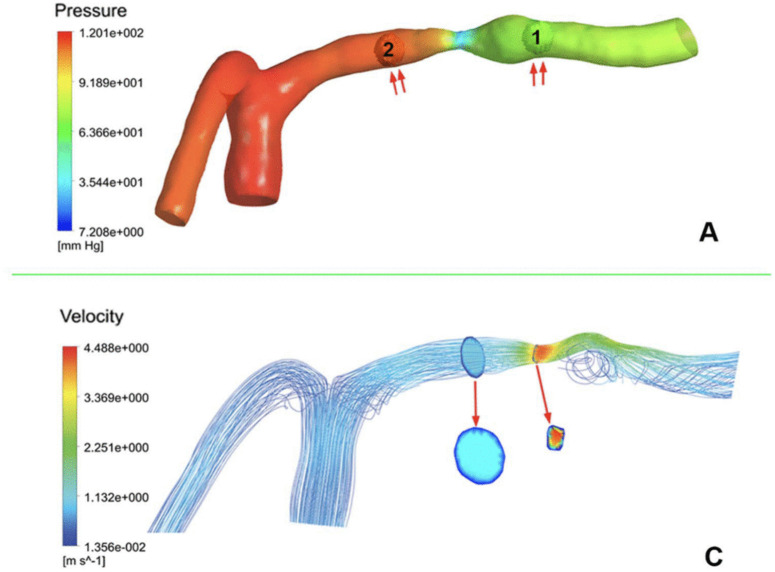
Pressure (A) and velocity (C) distributions in a stenotic intracranial artery. Adapted from Computational Fluid Dynamics Modeling of Symptomatic Intracranial Atherosclerotic Disease to Predict Risk of Stroke Recurrence (Leng et al., PLOS ONE, 2022). Panels (A) and (C) were vertically rearranged to illustrate the pressure-velocity relationship.

To further support the reliability of the present simulations, the results were compared with previously published CFD studies based on patient-specific intracranial stenosis geometries derived from clinical imaging data (Leng et al., 2014) [30]. Although direct in vivo flow measurements were not available, the reported hemodynamic parameters showed good agreement in both magnitude and trend, with deviations within 10%. This provides indirect support for the physiological plausibility of the present model. for an intracranial stenosis case with 70% stenosis (Fig 5).

In the reference work, the stenosed intracranial vessel was reconstructed from CTA images, discretized using ANSYS ICEM-CFD with an unstructured tetrahedral mesh of approximately 5 × 10⁵ elements, and simulated under a rigid-wall, no-slip assumption with a pressure-driven condition (inlet pressure 120 mmHg) governed by the incompressible Navier–Stokes equations.

As summarized in Table 3. Comparison between the results of the present study and those reported in the reference literature., the relative differences between the present study and the reference results for key hemodynamic metrics (e.g., velocity ratio and pressure ratio) were within 10%, supporting the reliability of the numerical setup adopted in this work.

**Table 3:** Summary of computed hemodynamic parameters under different stenosis severities (30%, 50%, 70%, and 90%) and blood viscosity conditions (below-normal, normal, and high).

		Below normal viscosity				Normal viscosity				High viscosity			
		30%	50%	70%	90%	30%	50%	70%	90%	30%	50%	70%	90%
Outlet1	Pressure	13390	13450	13360	13430	13390	13430	13350	13410	13360	13370	13350	13660
	Velocity	1.745	1.855	1.86	1.83	1.673	1.721	1.717	1.713	1.335	1.382	1.4	1.399
Outlet2	Pressure	13350	13350	13330	13330	13340	13350	13330	13330	13340	13340	13330	13330
	Velocity	1.464	1.217	0.1567	0.008	1.356	1.09	0.1358	0.0056	1.075	0.6811	0.06942	0.002
ES	Pressure	15950	14500	15240	15300	14530	14560	15250	15300	14590	14700	15260	15310
	Velocity	1.638	1.52	0.5076	0.179	1.533	1.417	0.3374	0.05641	1.155	1.103	0.1654	0.005
PS	Pressure	14180	13860	14430	17480	14180	13880	14430	14840	14220	14010	14550	14880
	Velocity	1.427	1.539	1.393	0.7995	1.377	1.505	1.269	0.6064	1.198	1.283	0.7377	0.2148
AS	Pressure	14000	13650	13590	13330	13990	13650	15320	13330	14000	13700	13420	13350
	Velocity	1.439	1.598	1.306	0.03143	1.495	1.528	1.033	0.02142	1.243	1.281	0.329	0.007
Stenosis part	WSS	102	83.93	86.79	107.3	109	98.41	92.12	107.2	128.7	86.01	102.5	98.41

### 3.1. Velocity distribution at outlet 1 and outlet 2

**Fig 6:**
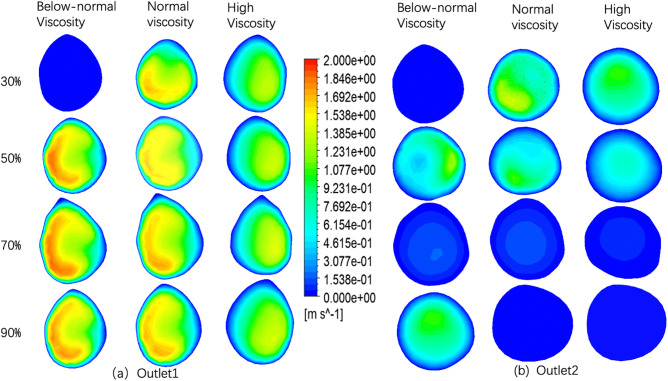
Velocity distributions at Outlet 1 (a) and Outlet 2 (b) under different stenosis severities (30%, 50%, 70%, and 90%) and blood viscosity conditions (below-normal, normal, and high).

The velocity profiles at Outlet 1 for four stenosis severities (30%, 50%, 70%, and 90%) under below-normal, normal, and high viscosity conditions are shown in Fig 6(a). With increasing stenosis severity, the profiles became progressively more asymmetric and off-center, indicating stronger downstream flow disturbance. At a given stenosis level, below-normal viscosity produced a more concentrated high-velocity core and steeper near-wall gradients, whereas high viscosity yielded smoother and more diffuse contours. Quantitatively, peak Outlet 1 velocity increased from 30% to 70% stenosis and then changed only slightly at 90% (Fig 10a andTable 4). The small difference between 70% and 90% stenosis (<0.03 m·s ⁻ ¹ across viscosity groups) suggests that further narrowing does not produce a proportional increase in distal branch velocity. Importantly, velocity at a given outlet does not necessarily vary monotonically with viscosity alone, because branch-wise flow is jointly influenced by viscosity, local geometry, stenosis-induced losses, and flow redistribution between outlets.

**Table 4:** TAWSS denotes time-averaged wall shear stress, defined as the mean magnitude of wall shear stress over one cardiac cycle. All values were obtained from transient simulations using the final cardiac cycle to ensure periodic stability. Different viscosity conditions (below-normal, normal, and high) represent inter-individual variability in blood rheology.

Stenosis(%)	Low-viscosity TAWSS (Pa)	Normal-viscosity TAWSS(Pa)	High-viscosity TAWSS(Pa)
30	37.64	49.48	106.2
50	41.50	53.87	117.6
70	44.80	59.37	133.1
90	33.82	45.27	98.04

**Fig 7:**
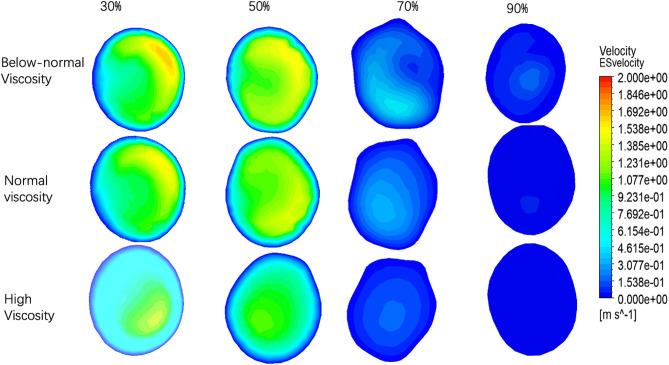
Velocity distributions at the early-stenotic section (ES) under different stenosis severities (30%, 50%, 70%, and 90%) and blood viscosity conditions (below-normal, normal, and high).

**Fig 8:**
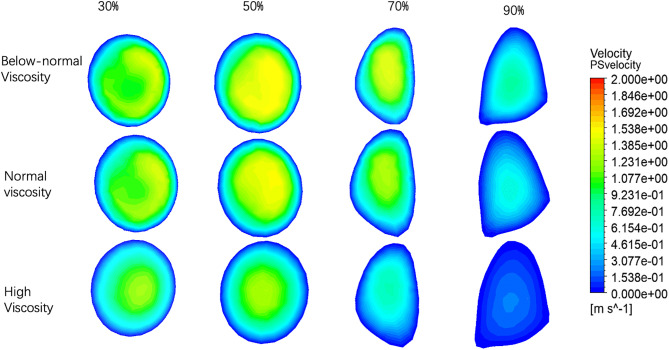
Velocity patterns at peak stenosis (PS) under varying stenosis ratios and viscosity conditions.

**Fig 9:**
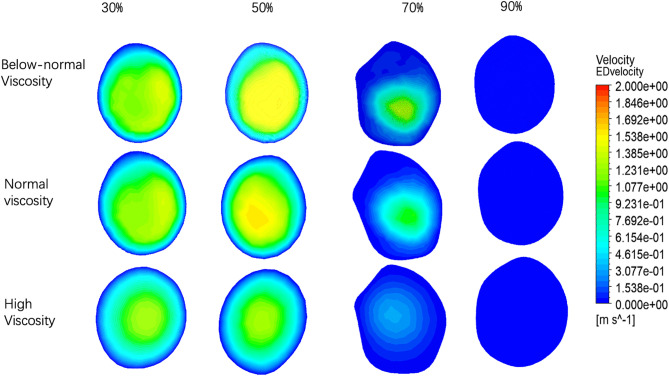
Blood flow velocity at the after stenosis (AS) site under varying stenosis ratios and viscosity conditions.

**Fig 10:**
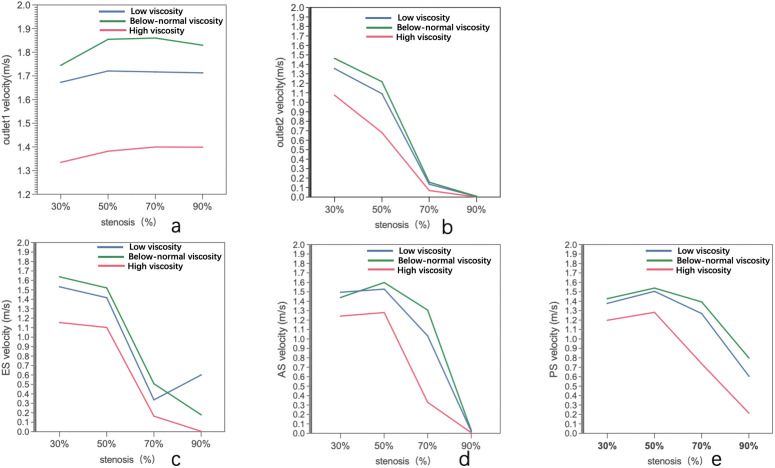
Velocity variations under below-normal, normal, and high blood viscosity conditions as functions of stenosis severity (30%, 50%, 70%, and 90%). **(a)** Velocity at Outlet 1, illustrating viscosity-dependent modulation of flow magnitude under moderate stenosis. **(b)** Velocity at Outlet 2, showing a sharp velocity collapse beyond 70% stenosis, indicating severe downstream flow limitation. **(c)** Velocity at the early-stenotic section (ES), highlighting the transition from acceleration-dominated to resistance-dominated flow regimes. **(d)** Velocity at the peak stenotic section (PS), where peak velocities are observed at moderate stenosis and decline markedly under critical narrowing. **(e)** Velocity at the after-stenotic section (AS), demonstrating pronounced flow deceleration and near-flow arrest at 90% stenosis.

The velocity profiles at Outlet 2 are shown in Fig 6(b). Compared with Outlet 1, Outlet 2 remained more organized and closer to axisymmetric, indicating greater downstream flow recovery along this branch. However, peak Outlet 2 velocity decreased markedly with stenosis severity (Fig 10b andTable 4). For below-normal viscosity, it declined from 1.464 m·s ⁻ ¹ at 30% stenosis to 1.217 m·s ⁻ ¹ at 50%, then dropped sharply to 0.1567 m·s ⁻ ¹ at 70% and 0.007945 m·s ⁻ ¹ at 90%. Similar trends were observed under normal and high viscosity conditions. Overall, Outlet 2 showed a biphasic decline, with modest reduction from 30% to 50% stenosis followed by pronounced collapse beyond 70% stenosis. This pattern indicates severe branch-wise flow redistribution and distal flow limitation as stenosis becomes critical, rather than a simple monotonic viscosity effect the velocity distributions at the ES across four stenosis severities (30%, 50%, 70%, and 90%) and three viscosity conditions (below-normal, normal, and high) shown in (Fig 7).

### 3.2. Velocity distribution at ES, PS, AS

At 30% stenosis, all cases exhibited a largely organized profile characterized by a central high-velocity core and a smooth peripheral transition. Viscosity primarily affected the compactness of the core: below-normal viscosity produced a more concentrated core with steeper gradients, whereas high viscosity yielded a more diffuse distribution.

At 50% stenosis, the ES profiles became increasingly asymmetric, with the below-normal viscosity case showing the most pronounced core elongation and eccentricity, while high viscosity maintained the most symmetric profile and a broader transition region.

At 70% stenosis, deformation of the ES velocity field was clearly enhanced. The below-normal viscosity condition exhibited the strongest asymmetry, with an off-center high-velocity region and sharp gradients; normal viscosity showed a similar spatial pattern with reduced magnitude, whereas high viscosity partially attenuated the asymmetry and produced comparatively smoother contours.

At 90% stenosis, ES velocities were low for all viscosity conditions, and the velocity field tended to appear more axisymmetric, particularly under normal and high viscosity, consistent with stronger viscous diffusion relative to inertial effects under low-velocity conditions.

Quantitative ES velocities are summarized in (Fig 10c and Table 4). The relative separation between viscosity groups increased with stenosis severity: at 30% stenosis, the below-normal viscosity case exceeded the high-viscosity case by 41.8% (1.638 vs 1.155 m·s ⁻ ¹), and at 50% stenosis by 37.8% (1.520 vs 1.103 m·s ⁻ ¹). This relative difference increased to 207.0% at 70% stenosis (0.5076 vs 0.1654 m·s ⁻ ¹) and 297.0% at 90% stenosis (0.1794 vs 0.0500 m·s ⁻ ¹). Notably, despite the increasing relative differences, the absolute velocity separation decreased as stenosis approached the critical level (0.483 m·s ⁻ ¹ at 30% vs 0.175 m·s ⁻ ¹ at 90%), reflecting an overall collapse of pre-stenotic velocities across all viscosity conditions.

The velocity distributions at the peak stenosis section (PS) for four stenosis severities (30%, 50%, 70%, and 90%) under below-normal, normal, and high viscosity conditions are shown in Fig 8. Overall, increasing stenosis severity led to progressively stronger distortion and asymmetry of the velocity profile at the stenotic throat, while viscosity mainly affected the compactness of the high-velocity core and the steepness of near-wall gradients.

At 30% stenosis, the PS profiles remained relatively organized for all viscosity conditions, with below-normal viscosity producing a steeper gradient and a more concentrated core, whereas high viscosity yielded a broader and more diffuse distribution. At 50% stenosis, profile deformation and eccentricity became more apparent, particularly under below-normal viscosity. At 70% stenosis, pronounced asymmetry was observed across all viscosity conditions, characterized by a skewed high-velocity region and an expanded low-velocity area on the opposite side, consistent with strong jet deflection near the throat. At 90% stenosis, the PS profiles became highly heterogeneous, with fragmented high-velocity regions embedded within broader low-velocity zones.

PS velocities are summarized in (Fig 10d and Table 4). In mild-to-moderate stenosis (30–50%), the below-normal viscosity condition exceeded the high-viscosity condition by approximately 19.1–19.9%, whereas viscosity-related differences became more pronounced at severe stenosis (70–90%). However, despite the larger relative differences, the absolute separation decreased as the overall velocity level declined. Peak PS velocity decreased markedly from 70% to 90% stenosis across all viscosity conditions, indicating that further narrowing beyond 70% did not produce proportional velocity amplification at the stenotic throat under the present modeling framework. This behavior suggests increasing flow limitation under near-critical stenosis, with the magnitude of decline becoming more pronounced at higher viscosity.

The velocity distributions at the AS across stenosis severities (30%, 50%, 70%, and 90%) and viscosity conditions (below-normal, normal, and high)Fig 9. At 30–50% stenosis, the AS velocity field remained relatively uniform, with broadly distributed medium-to-high velocities across the cross-section and only limited low-velocity regions near the wall. Under these mild-to-moderate stenosis conditions, viscosity primarily modulated velocity magnitude, while the overall spatial pattern remained similar among the three viscosity groups.

At 70% stenosis, the AS velocity field became markedly heterogeneous and asymmetric. Under normal viscosity, an expanded low-velocity region emerged on one side of the cross-section while the opposite side retained higher velocities. This heterogeneity was more pronounced for high viscosity, where low-velocity regions occupied a larger portion of the section and only a narrow residual flow channel remained, indicating that viscosity influenced not only velocity magnitude but also the spatial organization of post-stenotic flow at severe narrowing.

At 90% stenosis, velocities at the AS section approached near-zero values for all viscosity conditions, with only a very limited residual high-velocity core visible in the below-normal and normal viscosity cases. This pattern is consistent with a strong flow limitation under near-critical geometric constriction in the present pressure-driven setting.

AS velocities are summarized in (Fig 10e and Table 4). At 30% stenosis, peak AS velocity ranged from 1.243 to 1.495 m·s⁻¹, with the below-normal viscosity condition being 20.3% higher than the high-viscosity condition. At 50% stenosis, peak AS velocity increased slightly (1.281–1.598 m·s⁻¹) and the viscosity-related difference increased to 24.7%. A clear transition occurred at 70% stenosis: peak AS velocity decreased from 1.582 to 1.033 m·s⁻¹ under normal viscosity (−34.7%), and from 1.281 to 0.329 m·s⁻¹ under high viscosity (−74.3%). Correspondingly, the relative separation between below-normal and high viscosity widened sharply (from 24.7% at 50% to 297% at 70%). At 90% stenosis, peak AS velocities collapsed to 0.007–0.031 m·s⁻¹ across all viscosity conditions, representing a 97.6–97.9% reduction relative to 70% stenosis, with velocities converging toward near-zero values.

### 3.3. Pressure distribution at outlet 1 and 2 plane

**Fig 11:**
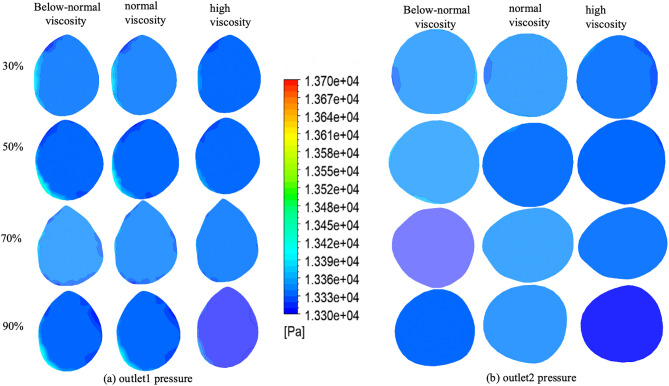
Pressure distribution at outlet 1 (a) and outlet 2 (b) under varying stenosis ratios and viscosity conditions.

Pressure distributions at Outlet 1 for four stenosis severities (30%, 50%, 70%, and 90%) and three viscosity conditions (below-normal, normal, and high) Fig 11a.

At 30% stenosis, the outlet pressure field was nearly uniform with minimal spatial variation across all viscosity conditions. At 50% stenosis, spatial heterogeneity became more apparent, most notably under below-normal viscosity, whereas high viscosity maintained comparatively smoother pressure contours. At 70% stenosis, a clear asymmetric pattern emerged for all viscosity conditions, indicating persistent downstream disturbance; high viscosity produced a similar distribution with a slightly smoother transition. At 90% stenosis, pronounced pressure inhomogeneity was observed in all cases, and viscosity-dependent differences in spatial variation became less distinct relative to the dominant geometric effect.

Outlet 1 pressure remained tightly clustered for 30–70% stenosis across viscosity conditions, ranging from approximately 13,350–13,450 Pa (≤100 Pa, ~ 0.75% variation). In contrast, at 90% stenosis, viscosity-related differences became more evident: the high-viscosity case reached 13,660 Pa, which was 230 Pa (1.71%) and 250 Pa (1.86%) higher than the below-normal and normal viscosity cases, respectively, suggesting increased hydraulic resistance under near-critical narrowing(Fig 15a andTable 4).

Fig 11(b) show the pressure distributions at Outlet 2 for four stenosis severities (30%, 50%, 70%, and 90%) and three viscosity conditions (below-normal, normal, and high). Overall, Outlet 2 exhibited a more spatially uniform pressure field than Outlet 1.

At 30–50% stenosis, mild pressure asymmetry and radial gradients were observed under below-normal and normal viscosity, whereas the high-viscosity condition maintained comparatively smoother and more uniform contours, consistent with stronger downstream viscous dissipation. At 70% stenosis, pressure heterogeneity further diminished compared with Outlet 1, and the cross-sectional distributions remained relatively ordered.At 90% stenosis, the pressure field at Outlet 2 became nearly uniform across all viscosity conditions, indicating that downstream pressure differences were largely attenuated under near-critical flow reduction in the present boundary-condition setting.

Quantitative values are summarized in (Fig 15b and Table 4). Outlet 2 pressure remained tightly clustered across all stenosis and viscosity conditions, ranging from 13,330–13,350 Pa (≤20 Pa, ~ 0.15% variation). Within each viscosity group, pressure changes with stenosis were minimal (below-normal: 13,350 Pa at 30–50% and 13,330 Pa at 70–90%; normal: 13,330–13,350 Pa; high: 13,330–13,340 Pa). These results suggest that Outlet 2 pressure is primarily governed by the downstream boundary condition and exhibits limited sensitivity to upstream stenosis severity or viscosity within the present modeling framework.

### 3.4. Pressure distribution at ES, PS, AS

**Fig 12:**
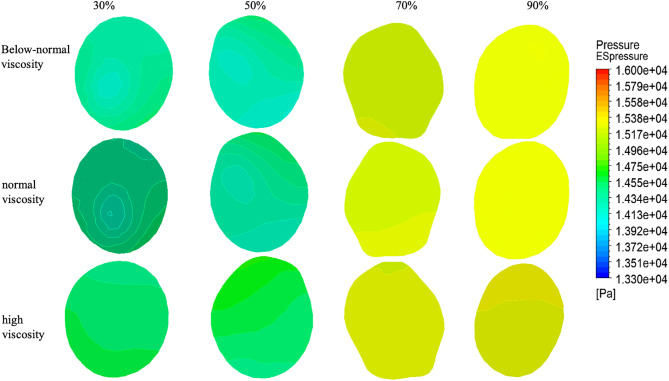
Pressure distribution at the early stenosis (ES) section under varying stenosis ratios and viscosity conditions.

Pressure distributions at the ES cross-section for four stenosis severities (30%, 50%, 70%, and 90%) under three viscosity conditions (below-normal, normal, and high) Fig 12.

At 30% stenosis, the ES pressure field exhibited viscosity-dependent spatial heterogeneity, with the below-normal viscosity case showing the largest cross-sectional variation, whereas high viscosity produced the smoothest and most uniform distribution. At 50% stenosis, pressure non-uniformity became more apparent, particularly for below-normal and normal viscosity, while the high-viscosity case maintained comparatively smoother contours.At 70% stenosis, an asymmetric pressure pattern was observed for all viscosity conditions, with expanded low-pressure regions and localized higher-pressure zones on the opposite side. At 90% stenosis, the ES pressure field remained heterogeneous; however, the cross-sectional patterns across viscosity groups became more similar, consistent with the dominant influence of severe geometric constriction.

ES pressures are summarized in (Fig 15c and Table 4). At 30% stenosis, the below-normal viscosity condition showed the highest ES pressure (15,950 Pa), exceeding normal viscosity by 9.8% and high viscosity by 9.3%. At 50% stenosis, the below-normal viscosity case exhibited the lowest ES pressure (14,500 Pa), differing only slightly from the normal- and high-viscosity cases. At higher stenosis severities (70–90%), ES pressure values converged across viscosity conditions: 15,240–15,260 Pa at 70% stenosis and 15,300–15,310 Pa at 90% stenosis (≤70 Pa difference, < 0.5%). This convergence indicates reduced sensitivity of ES pressure to viscosity as stenosis approaches critical narrowing under the present resistance-informed boundary setting.

**Fig 13:**
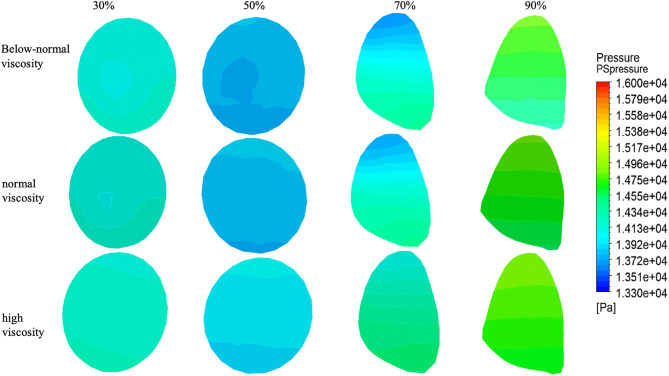
Pressure distribution at the peak stenosis (PS) section under varying stenosis ratios and viscosity conditions.

Fig 13 show the pressure distributions at the PS for four stenosis severities (30%, 50%, 70%, and 90%) under three viscosity conditions (below-normal, normal, and high).

At 30% stenosis, the PS pressure field remained relatively ordered for all viscosity conditions, characterized by a localized low-pressure region and a surrounding higher-pressure zone; below-normal and normal viscosity produced sharper spatial gradients, whereas high viscosity exhibited smoother transitions. At 50% stenosis, pressure inhomogeneity increased in all cases, with a more concentrated low-pressure core and steeper gradients, most pronounced under below-normal viscosity. At 70% stenosis, the pressure field became strongly asymmetric, showing spatial segregation of low- and high-pressure regions across the cross-section; viscosity mainly influenced the sharpness of the transition rather than the overall asymmetry pattern. At 90% stenosis, the PS pressure distributions were highly heterogeneous for all viscosity conditions, with fragmented extrema and irregular contours, while differences among viscosity groups became less distinct compared with the dominant geometric effect at near-critical narrowing.

PS showed limited viscosity dependence under mild stenosis (30–50%), remaining within approximately 14,010–14,220 Pa across conditions (Fig 15d and Table 3). In contrast, at 90% stenosis, a marked viscosity-related separation was observed: the below-normal viscosity case reached 17,480 Pa, which was 17.8% and 17.5% higher than the normal- and high-viscosity cases, respectively. This result indicates that, under near-critical stenosis, pressure at the throat becomes more sensitive to viscosity within the present pressure-driven modeling framework.

**Fig 14:**
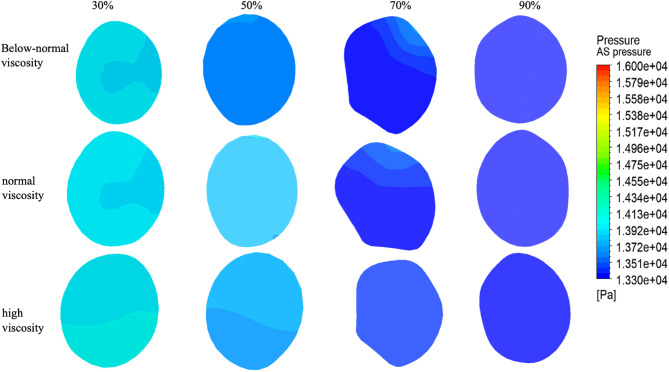
Pressure distribution at the AS section under varying stenosis ratios and viscosity conditions.

Fig 14 show the pressure distributions at the AS across stenosis severities (30%, 50%, 70%, and 90%) and viscosity conditions (below-normal, normal, and high).

At 30–50% stenosis, the AS pressure field exhibited a relatively regular spatial gradient and remained qualitatively similar across viscosity groups, indicating limited viscosity influence on the overall downstream pressure pattern under mild-to-moderate narrowing.

At 70% stenosis, the AS pressure field became markedly asymmetric, most evident under the normal-viscosity condition, where a large low-pressure region occupied one side of the cross-section while higher-pressure regions remained on the opposite side.

At 90% stenosis, the AS pressure field showed a localized pressure concentration with a surrounding lower-pressure region across viscosity conditions, consistent with strong flow limitation under near-critical narrowing.

**Fig 15:**
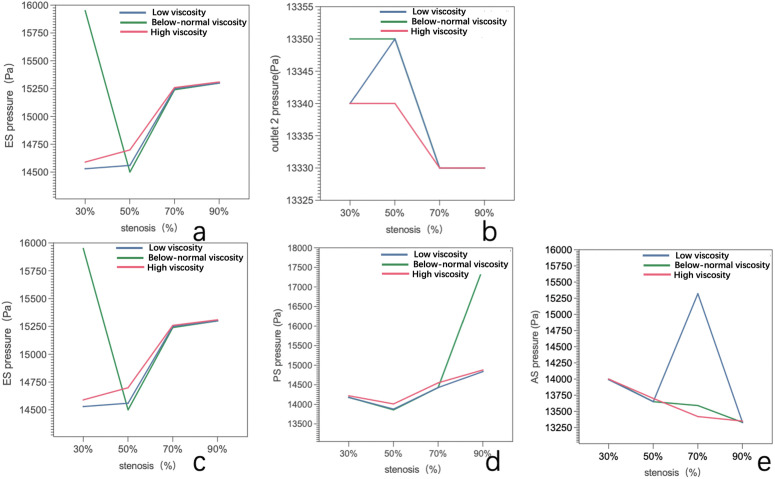
Pressure variations under below-normal, normal, and high blood viscosity conditions as functions of stenosis severity (30%, 50%, 70%, and 90%). **(a)** Pressure at Outlet 1, showing viscosity-dependent pressure drop and increased pressure gradient demand under severe stenosis. **(b)** Pressure at Outlet 2, demonstrating near-uniform pressure levels across stenosis severities, indicating dominant influence of downstream boundary conditions. **(c)** Pressure at the early stenosis (ES) section, highlighting upstream pressure accumulation and the transition to resistance-dominated flow beyond moderate stenosis. **(d)** Pressure at the peak stenosis (PS) section, where pronounced pressure reduction is observed at critical stenosis, particularly under elevated viscosity conditions. **(e)** Pressure at the after stenosis (AS) section, illustrating pressure dissipation and non-monotonic behavior associated with flow reorganization downstream of severe narrowing.

Quantitative AS pressures are summarized in (Fig 15e and Table 3). In most stenosis–viscosity combinations, AS pressure decreased with increasing stenosis, from approximately 14,000 Pa at 30% to ~13,650 Pa at 50% and ~13,330 Pa at 90%. An exception was observed at 70% stenosis under normal viscosity, where AS pressure increased to 15,320 Pa (9.4% higher than 30% and 14.9% higher than 90%). This localized pressure elevation coincided with the stenosis severity at which velocities began to decline substantially, providing additional evidence of a transition in the flow regime near 70% stenosis under the present resistance-informed boundary setting.

### 3.5. Wall shear stress distribution at the stenosis region

**Fig 16:**
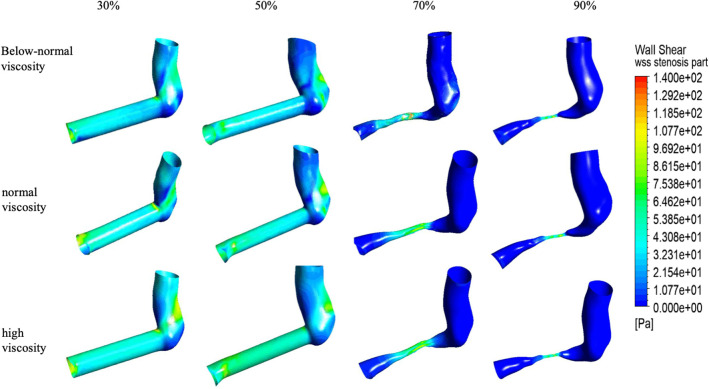
Wall shear stress (WSS) distribution on the stenosed artery under varying stenosis ratios and viscosity conditions.

The wall shear stress (WSS) distributions along the stenosis region under four stenosis severities (30%, 50%, 70%, and 90%) and three viscosity conditions (below-normal, normal, and high)(Fig 16). Across all cases, elevated WSS was concentrated within the stenosis segment, whereas the AS expansion region exhibited extensive low-WSS areas that became more prominent with increasing stenosis severity.

At 30% stenosis, WSS remained relatively low and spatially uniform upstream of the lesion, while a localized high-WSS band developed within the stenosis region. The WSS within the stenosis region reached approximately 102 Pa (below-normal), 109 Pa (normal), and 127 Pa (high viscosity), followed by a gradual downstream decay.

At 50% stenosis, high-WSS localization within the stenosis region became more pronounced, with steeper spatial gradients, and the AS region displayed alternating patches of higher and lower WSS consistent with developing disturbed-flow structures.

At 70% stenosis, WSS within the stenosis region remained elevated (approximately 80.8–102.5 Pa, depending on viscosity), while a pronounced and spatially extensive low-WSS zone dominated the post-stenotic region, indicating strong flow separation and reduced near-wall shear over a large downstream area. At 90% stenosis, WSS heterogeneity was most pronounced: WSS within the stenosis region was approximately 107 Pa (below-normal), 107 Pa (normal), and 98.4 Pa (high viscosity), while the AS region was largely characterized by very low WSS, with only limited areas of moderate WSS where residual jets impinged on the wall.

**Fig 17:**
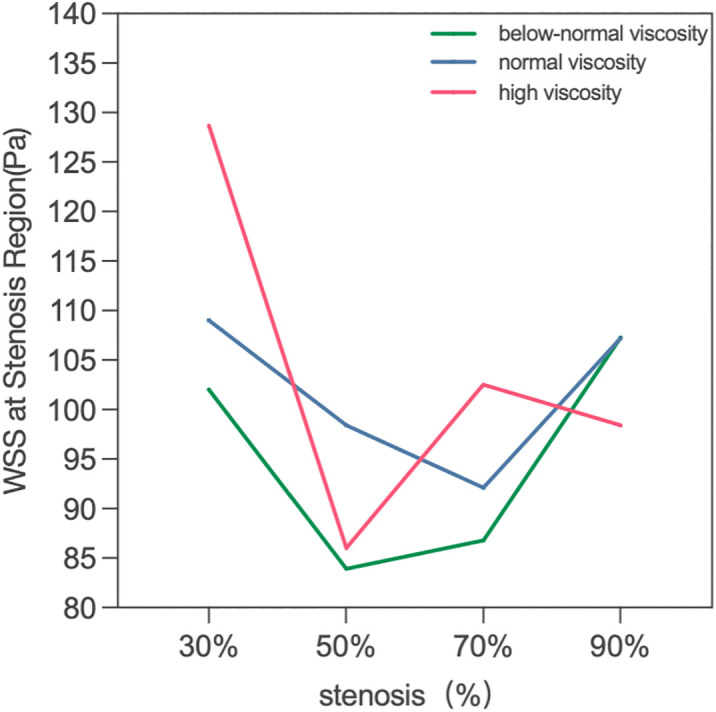
Peak wall shear stress (WSS) within the stenosis region under varying stenosis ratios and viscosity conditions.

WSS values within the stenosis region are summarized in (Fig 17). At 30% stenosis, WSS increased with viscosity (102.0 Pa < 109.0 Pa < 128.7 Pa), with the high-viscosity case being 26.2% higher than the below-normal case. With increasing stenosis severity, viscosity-related differences in WSS became less pronounced. At 70–90% stenosis, WSS values clustered within a relatively narrow range (approximately 86–107 Pa). At 90% stenosis the three viscosity conditions differed by <9% (107.3 Pa, 107.2 Pa, and 98.4 Pa for below-normal, normal, and high viscosity, respectively). This convergence suggests that, under severe narrowing, WSS within the stenosis region becomes increasingly governed by geometry-induced near-wall velocity gradients, whereas viscosity plays a secondary role in determining the peak value.

Notably, WSS within the stenosis region did not increase monotonically with stenosis severity (range 83.9–128.7 Pa across all cases), indicating that WSS alone may not fully discriminate stenosis severity in this modeling framework. In contrast, the spatial extent and persistence of low-WSS regions downstream of the stenosis increased markedly from 70% to 90% stenosis, providing complementary hemodynamic information beyond peak values.

### 3.6. Mean wall shear stress (TAWSS) distribution

TAWSS is a key parameter to detect critical locations where atherosclerosis is prone to rupture. TAWSS is calculated using the following formula: $TAWSS=\frac{\text{1}}{T}\;(\int_{\text{0}}^{T}\left|T_{w}\right|\text{dt})$. TAWSS represents the time average value of wall shear stress in a cardiac cycle and is used to indicate the average shear load level on the vessel wall. In general, with the increase of stenosis degree, the hemodynamic load gradually changes from relatively scattered to highly concentrated in the local stenosis segment, especially in the 90% stenosis.

**Fig 18:**
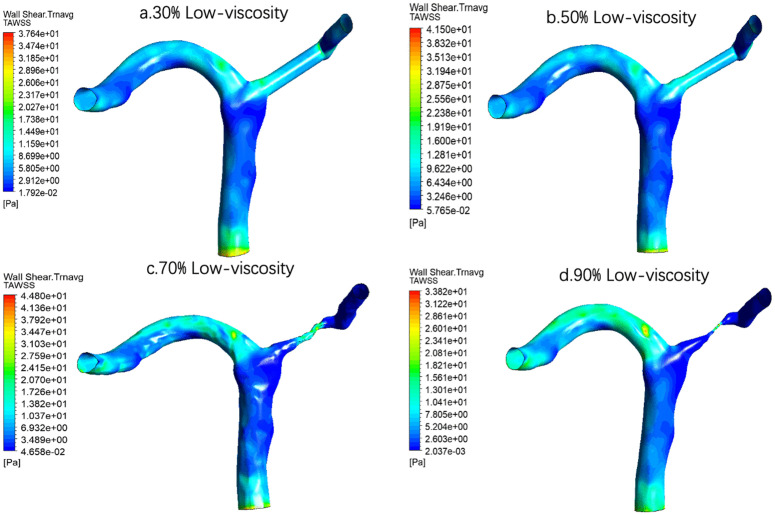
Time-averaged wall shear stress (TAWSS) distribution under low-viscosity conditions across varying stenosis severities: (a) 30%, (b) 50%, (c) 70%, and (d) 90%. Color scale indicates TAWSS magnitude in Pa, with elevated shear stress observed at the stenosis throat progressively increasing with severity.

For low-viscosity conditions Fig 18, at 30% stenosis, the overall distribution is relatively uniform, and only a few elevated areas are visible around the lateral curvature of the vessel, near the bifurcation, and some exits. The 50% stenosis is similar to the 30% stenosis, but the local inhomogeneity begins to enhance. However, the obvious change of TAWSS distribution at 70% is a very critical turning point. A more concentrated high-speed zone appears in the stenosed segment, and blood flow is significantly accelerated at the stenosed segment, resulting in a stronger shear stress level on the wall. At the same time, the non-stenosed region is still dominated by lower TAWSS, indicating that a strong spatial gradient has been formed in the vessel at this time. This suggests that the hemodynamics at 70% stenosis has changed from an overall slow disturbance under mild-to-moderate stenosis to an apparent local high-speed jet dominant pattern. This local centralization tendency is more prominent at 90% stenosis. This indicates that under severe stenosis, the blood flow is strongly compressed through the narrow throat, resulting in local high-speed jet, resulting in significantly higher TAWSS in a very small range. However, the average shear level is relatively low in the post-stenosis and other wall regions due to the effects of restricted flow distribution, flow separation, or low-speed recirculation.

**Fig 19:**
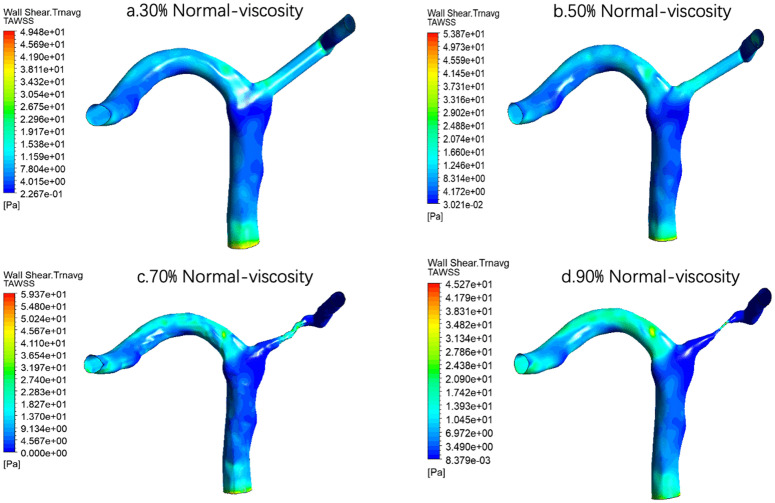
Time-averaged wall shear stress (TAWSS) distribution under normal-viscosity conditions across varying stenosis severities: (a) 30%, (b) 50%, (c) 70%, and (d) 90%. Color scale indicates TAWSS magnitude in Pa, demonstrating progressive elevation of peak shear stress at the stenosis throat with increasing severity.

For normal viscosity, the overall trend of TWASS spatial distribution was consistent with that under low viscosity condition, and TAWSS concentrated locally in stenosis with the aggravation of stenosis Fig 19. However, under normal viscosity, the local high TAWSS area was more clearly displayed at 70% and 90% stenosis, suggesting that the higher viscosity may further strengthen the local shear stress level of the stenosis segment.

**Fig 20:**
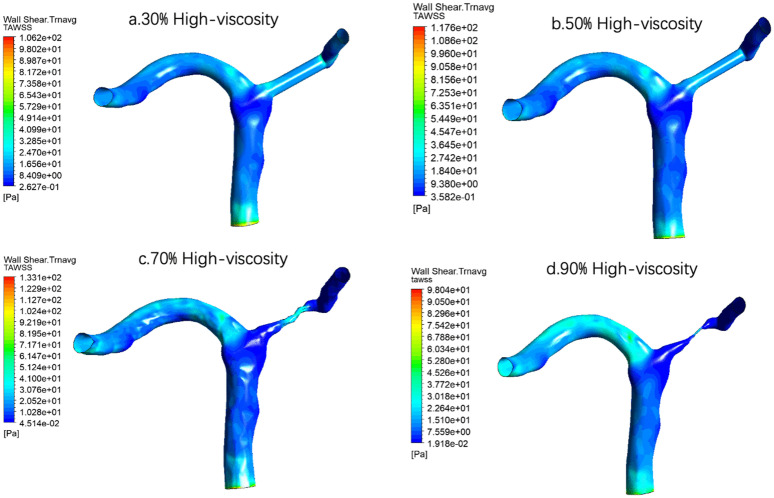
Time-averaged wall shear stress (TAWSS) distribution under high-viscosity conditions across varying stenosis severities: (a) 30%, (b) 50%, (c) 70%, and (d) 90%. Color scale indicates TAWSS magnitude in Pa, demonstrating the most pronounced peak shear stress elevation at the stenosis throat among all viscosity conditions.

Under the condition of high viscosity Fig 20, the spatial distribution trend of TAWSS was still consistent, that is, with the increase of stenosis degree from 30% to 70%, the high TAWSS area in the stenosis area gradually enhanced and became more concentrated. However, when the stenosis was further aggravated to 90%, TAWSS did not continue to increase, but showed varying degrees of decline. This indicates that under very severe stenosis, although there may still be a very limited high-shear area in the stenosis site, the overall flow capacity is reduced and the flow limitation is aggravated, which weakens the time-averaged shear effect on a wide range of walls. The relationship between TAWSS and stenosis degree is not a simple monotonic increase, but is more likely to show a change pattern of first increasing and then decreasing, in which 70% stenosis may correspond to an important hemodynamic turning point.

**Fig 21:**
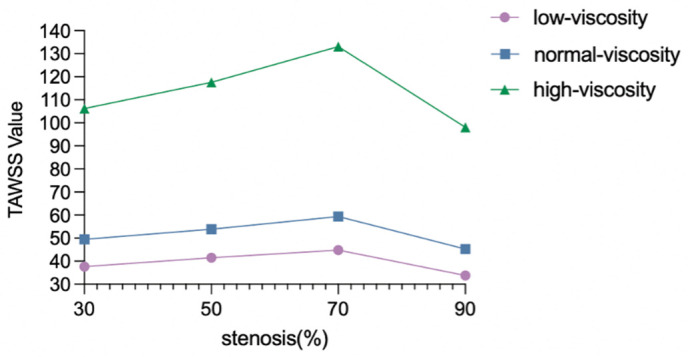
Time-averaged wall shear stress (TAWSS) as a function of stenosis severity for low-, normal-, and high-viscosity conditions. TAWSS increases progressively with stenosis severity up to 70%, followed by a decline at 90% stenosis due to flow limitation. The viscosity-dependent amplification is most pronounced at 70% stenosis, where high-viscosity conditions produce TAWSS values approximately 3-fold higher than low-viscosity conditions.

For example, the quantitative results showed that at the same degree of stenosis, the increase of blood viscosity was generally correlated with the increase of TAWSS, and the TAWSS level of the high viscosity group was generally higher than that of the normal viscosity group and the low viscosity group(Table 4 and Fig 21). This phenomenon suggests that the enhanced blood viscosity amplifies the time-averaged effect of wall shear stress, thereby reinforcing the high-shear environment in the stenosis region. However, the basic pattern of TAWSS change with stenosis degree was not changed by increasing viscosity. Under all three viscosity conditions, TAWSS showed a gradual increase from 30% to 70% stenosis, and reached the most obvious at 70% stenosis, while falling back at 90% stenosis. The results suggest that the viscosity mainly determines the absolute level of TAWSS, while the degree of stenosis determines its evolution trend. In severe but not yet fully confined stages, local flow acceleration dominates the increase in TAWSS, while in extremely severe stenosis conditions, global flow restriction may offset or even exceed the shear enhancement effect of increased viscosity.

### 3.7. Shear oscillator (OSI) distribution

OSI is another key hemodynamic parameter, which is between 0 and 0.5, where 0 indicates a completely unidirectional flow shear environment, and close to 0.5 indicates that the direction of wall shear stress has a very strong round trip reversal in a cycle, and the positive and negative directions are close to cancel each other.

**Fig 22:**
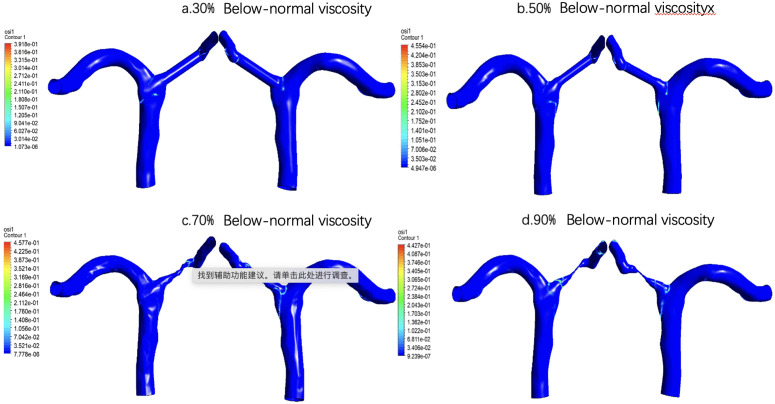
Oscillatory shear index (OSI) distribution under low-viscosity conditions across varying stenosis severities: (a) 30%, (b) 50%, (c) 70%, and (d) 90%. Color scale indicates OSI magnitude (0–0.5), with elevated values indicating flow recirculation and oscillation. Minimal OSI elevation is observed under low-viscosity conditions, suggesting predominantly unidirectional flow even at severe stenosis.

This figure demonstrates the OSI distribution characteristics of models with different degrees of stenosis in a low viscosity environment Fig 22. At 30% stenosis, OSI was lower in most areas of the vessel wall, and only a small amount of mild elevation occurred near the top of the bifurcation and stenosis-related pathways. This indicates that the near-wall blood flow direction is still relatively stable under mild stenosis, and the mechanical stimulation in most endothelial areas is relatively smooth, with only limited oscillatory flow in the local area. At this time, the whole vessel wall is still dominated by a relatively stable flow microenvironment. At 50% stenosis, with the aggravation of stenosis, the local blood flow direction began to oscillate significantly, and the stability of mechanical stimulation to the endothelium decreased. When the stenosis was increased to 70%, the OSI was significantly higher and a more continuous and prominent area of high value was formed in the stenosis segment and its adjacent wall. This means that the endothelium in these areas is no longer primarily subjected to stable unidirectional shear, but is chronically exposed to mechanical stimuli with more pronounced directional changes. Such oscillatory blood flow environment is generally considered to be detrimental to the maintenance of endothelial function and may promote inflammation, abnormal endothelial function and adverse remodeling of the local vascular wall. However, at 90% stenosis, OSI decreased compared to 70%. Although a local elevation area was still visible near the stenosis, the overall range and intensity of the elevation were weaker than that of the 70% stenosis, indicating that the oscillatory shear environment exposed to the endothelium was not further enhanced but rather weakened under extremely severe stenosis. That is to say, 90% stenosis is not the strongest stage of oscillatory flow, but more likely to show that under severe flow limiting conditions, the local flow regime changes, so that the round-trip swing in the direction of wall shear stress is no longer as significant as in 70% stenosis.

**Fig 23:**
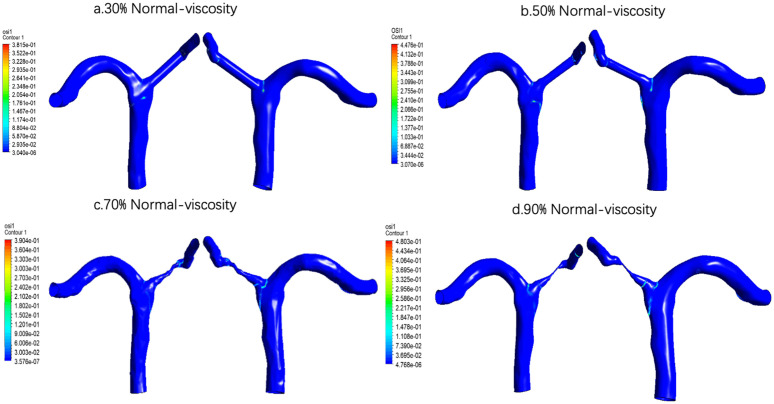
Distribution of oscillatory shear index (OSI) under normal viscosity conditions for different stenosis severities: (a) 30%, (b) 50%, (c) 70%, and (d) 90%. Increased stenosis severity leads to the development and expansion of regions with elevated OSI, particularly in the post-stenotic region, indicating enhanced flow disturbance and oscillatory behavior.

Compared with the low viscosity condition Fig 23, the overall trend of OSI under normal viscosity condition is the same and most of them have obvious differences. At low viscosity, OSI is most obvious when the stenosis degree increases to 70%, and decreases when the stenosis degree is 90%. On the contrary, under normal viscosity conditions, OSI already increased significantly at 50% stenosis, although it fell back slightly at 70% stenosis, but it increased again and reached the highest level at 90% stenosis. This suggests that elevated blood viscosity not only affects the strength of the oscillatory shear environment, but may also alter its distribution pattern at different stages of stenosis. In terms of physiological significance, extremely severe stenosis at normal viscosity is more likely to expose the local endothelium to strong oscillatory disturbed flow microenvironment, while this adverse environment is most prominent at 70% stenosis stage under low viscosity conditions.

**Fig 24:**
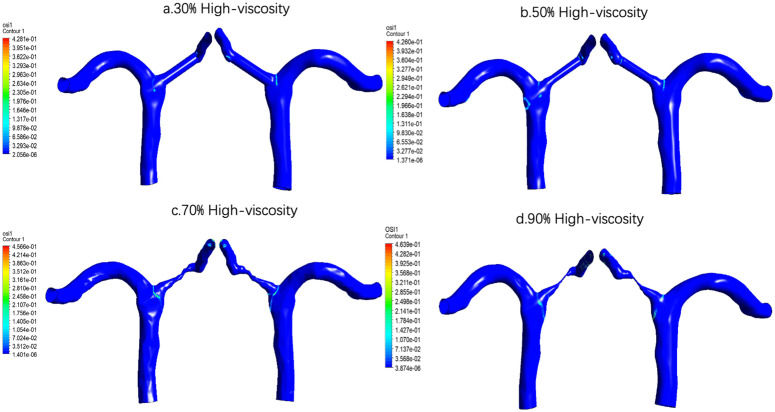
Spatial distribution of oscillatory shear index (OSI) under high viscosity conditions for increasing stenosis severities: (a) 30%, (b) 50%, (c) 70%, and (d) 90%. Higher OSI values are primarily observed in the post-stenotic region, reflecting increased flow disturbance associated with severe narrowing.

In the high viscosity environment Fig 24, when 30% stenosis, most areas of the vessel wall are still dominated by low OSI. At 50% stenosis, OSI was not significantly enhanced compared with 30%, and the overall level remained low. When the stenosis increased to 70%, OSI increased significantly, and a more prominent distribution of high values appeared in the stenosis segment and adjacent areas, suggesting that the local endothelium began to be exposed to stronger directional shock stimulation. At 90% stenosis, OSI increased further and reached the highest level in this group. These results indicate that the oscillatory mechanical stimulation is the strongest in the local endothelium under very severe stenosis. In a physiological sense, this suggests that 90% stenosis is most likely to form an environment that is not conducive to the maintenance of endothelial homeostasis under high viscosity conditions.

**Fig 25:**
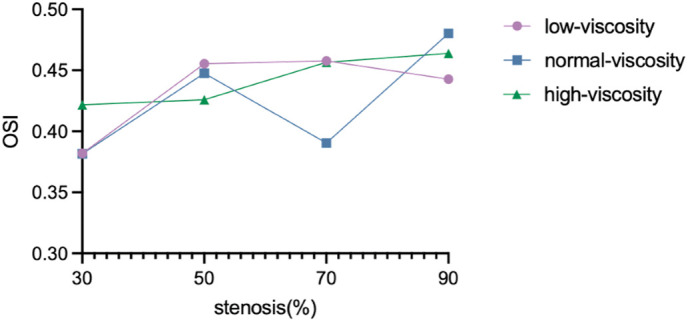
Variation of oscillatory shear index (OSI) with stenosis severity under different blood viscosity conditions (below-normal, normal, and high). OSI generally increases with increasing stenosis severity, indicating enhanced flow oscillation and disturbance, particularly in severe stenosis cases.

Under different viscosity conditions, the response pattern of OSI to the degree of stenosis was significantly different, suggesting that the local endothelial mechanical microenvironment evolved differently with the change of viscosity and stenosis severity(Fig 25 and Table 5). Under low viscosity conditions, OSI first increased and then decreased with the increase of stenosis degree, reaching the most obvious at 70% stenosis and decreasing at 90% stenosis, indicating that the oscillatory shear environment was most prominent at severe stenosis stage, but not further enhanced at extremely severe stenosis. In contrast, under normal viscosity conditions, OSI fluctuated nonmonotonically and reached its maximum at 90% stenosis, suggesting that the local endothelium was exposed to a more oscillatory disturbed flow microenvironment under extremely severe stenosis. Under high viscosity conditions, OSI overall increased with the increase of stenosis degree, and also reached the highest at 90% stenosis, indicating that higher viscosity could further enhance oscillatory shear stimulation under extremely severe stenosis conditions. In conclusion, the adverse oscillatory endothelial microenvironment under low viscosity conditions mainly appeared in the 70% stenosis stage, while under normal and high viscosity conditions, the adverse microenvironment was more concentrated in the 90% stenosis stage. This suggests that blood viscosity not only affects the amplitude level of OSI, but may also alter the stenotic stage of the most adverse oscillatory flow environment, thereby affecting the timing and extent of local endothelial homeostasis impairment.

**Table 5:** OSI denotes oscillatory shear index, which quantifies the directional oscillation of wall shear stress over a cardiac cycle. OSI ranges from 0 (unidirectional flow) to 0.5 (fully oscillatory flow). All values were obtained from transient simulations using the final cardiac cycle to ensure periodic stability. Different viscosity conditions (below-normal, normal, and high) represent inter-individual variability in blood rheology.

Stenosis(%)	Low-viscosity OSI (Pa)	Normal-viscosity OSI(Pa)	High-viscosity OSI(Pa)
30	0.3819	0.3815	0.4281
50	0.4554	0.4476	0.4260
70	0.4577	0.3904	0.4566
90	0.4427	0.4803	0.4639

## 4. Discussion

The present study employed a non-Newtonian Carreau model to investigate the coupled effects of stenosis severity and blood viscosity on intracranial hemodynamics within a patient-derived bifurcating geometry. Several key findings emerge, with potential implications for the hemodynamic assessment and clinical management of intracranial atherosclerotic disease (ICAD).

### 4.1. Hemodynamic transition near moderate-to-severe stenosis

A transition-like hemodynamic change was observed around 70% stenosis in the present model [31]. This transition is characterized by a shift from acceleration-dominated flow to resistance-dominated behavior, reflected by the non-monotonic variation of outlet velocity, the collapse of distal branch flow, and the redistribution of flow between outlets. This behavior may depend on multiple factors, including vessel geometry eccentricity, pulsatile flow conditions, downstream vascular resistance, and plaque morphology. From a clinical perspective, this suggests that stenosis severity alone may not linearly reflect downstream perfusion [32,33]. In particular, lesions approaching this range may exhibit disproportionately reduced distal flow despite only modest increases in geometric narrowing [34,35]. Such behavior may help explain why patients with similar angiographic stenosis percentages can present with markedly different perfusion status or stroke risk [36].

### 4.2. Coupled effects of stenosis severity and viscosity

Second, the results demonstrate that blood viscosity interacts with stenosis severity in a non-trivial manner. While viscosity primarily modulates flow magnitude under mild stenosis, it significantly alters flow organization, asymmetry, and recirculation patterns under severe stenosis [37,38]. Importantly, outlet velocities did not vary monotonically with viscosity due to flow redistribution between branches [39].This indicates that inter-individual differences in blood rheology (e.g., hematocrit variation or hyperviscosity states) may influence not only global flow resistance but also regional perfusion distribution [40,41]. Therefore, incorporating rheological variability may improve individualized hemodynamic risk assessment beyond purely geometric measures [42].

### 4.3. Pathological wall shear environment in severe stenosis

Dual pathological wall shear stress (WSS) pattern was identified under severe stenosis, characterized by elevated WSS within the stenotic throat and extensive low-WSS regions downstream [43,44]. High WSS has been associated with endothelial damage and plaque destabilization, whereas low WSS is linked to inflammatory activation, lipid accumulation, and thrombus formation [45,46]. In the present study, the downstream low-WSS region expanded markedly from 70% to 90% stenosis, suggesting that post-stenotic disturbed flow may be a critical contributor to disease progression [47]. This finding highlights that clinical risk assessment should consider not only peak stenotic severity but also downstream hemodynamic environments.

### 4.4. Added value of pulsatility-based metrics

The inclusion of pulsatility-based metrics further strengthens the clinical relevance of the present findings. TAWSS exhibited a non-monotonic trend, increasing up to moderate-to-severe stenosis and then decreasing under critical narrowing, indicating reduced overall wall shear loading due to flow limitation [48]. Meanwhile, OSI demonstrated a viscosity-dependent response, reflecting changes in flow oscillation and directional instability [49,50]. Clinically, this suggests that lesions with similar angiographic stenosis severity may still differ substantially in their downstream shear environment and hemodynamic risk. In particular, regions of reduced TAWSS and elevated OSI may indicate endothelial dysfunction, disturbed flow, and thrombus-prone conditions, thereby providing complementary information for identifying lesions at higher risk of progression or ischemic events [17,51].

### 4.5. Implications for hemodynamic assessment of ICAD

From a modeling perspective, the use of a non-Newtonian Carreau model is justified by the need to capture shear-dependent viscosity variations across the flow domain [52]. While Newtonian assumptions may be adequate in high-shear regions such as the stenotic throat, they may underestimate hemodynamic complexity in post-stenotic and near-wall regions where low shear and recirculation occur [53]. Given that clinically relevant metrics such as WSS, TAWSS, and OSI are highly sensitive to local flow conditions, non-Newtonian modeling provides a more comprehensive basis for hemodynamic interpretation, although Newtonian approximations may still be acceptable for certain global quantities [54].

Overall, the present findings suggest that hemodynamic evaluation of ICAD should move beyond purely geometric stenosis metrics and incorporate flow redistribution, rheological variability, and wall shear–related indicators. Such an approach may contribute to improved patient-specific risk stratification and support more informed clinical decision-making in the management of intracranial arterial stenosis.

### 4.6. Research limitations and future directions

Several limitations should be acknowledged. Although transient simulations were included for TAWSS and OSI, the wall was assumed rigid and the pulsatile input was not fully patient-specific. The geometry was derived from a patient-specific model, but the stenosis variants were generated as controlled simplified modifications and therefore do not fully reproduce plaque eccentricity or vascular remodeling. In addition, while the revised outlet treatment improved physiological realism, direct validation against patient-specific Doppler or in vivo flow measurements was not available. Although a non-Newtonian Carreau model was employed, a systematic sensitivity analysis of rheological model parameters was not performed, and formal uncertainty quantification was beyond the scope of the present study. Reynolds number analysis suggests predominantly laminar flow, potential localized transitional effects under severe stenosis were not explicitly investigated, and Womersley number analysis was not included.

Future studies should incorporate patient-specific pulsatile boundary conditions, vascular compliance, more realistic plaque morphology, direct clinical flow measurements, rheological parameter sensitivity analysis, uncertainty quantification, and additional pulsatile flow characterization to further refine hemodynamic prediction in ICAD.

## 5. Conclusion

In this study, a non-Newtonian Carreau model was used to evaluate the coupled effects of stenosis severity and blood viscosity on intracranial hemodynamics. The results show that flow behavior is governed by the combined influence of geometry and rheology, rather than by stenosis severity alone. A marked transition-like hemodynamic change was observed near 70% stenosis in the present model. In particular, Outlet 2 velocity decreased sharply from 1.217–0.681 m·s ⁻ ¹ at 50% stenosis to 0.157–0.069 m·s ⁻ ¹ at 70%, and further approached near-zero values (0.008–0.002 m·s ⁻ ¹) at 90%, indicating severe distal flow limitation and redistribution between branches.

Severe stenosis produced a dual pathological shear environment, characterized by elevated WSS within the stenotic region and disturbed low-shear flow downstream. Peak WSS in the stenotic segment ranged from approximately 83–129 Pa, while extensive low-WSS regions developed distal to the lesion. In addition, TAWSS showed a non-monotonic response, increasing up to 70% stenosis and then decreasing at 90% stenosis; for example, under high-viscosity conditions, TAWSS increased from 106.2 Pa at 30% stenosis to 133.1 Pa at 70%, then decreased to 98.04 Pa at 90%. OSI also showed viscosity-dependent elevation under severe stenosis, with values ranging from approximately 0.38 to 0.48, indicating enhanced oscillatory and disturbed flow.

Overall, these findings suggest that the hemodynamic assessment of intracranial atherosclerotic disease should extend beyond conventional geometric stenosis measures to include rheological variability and wall shear–based metrics such as WSS, TAWSS, and OSI. Such an integrated approach may improve patient-specific hemodynamic evaluation and provide more clinically meaningful information for risk stratification.

All figures and tables are provided in S1 Fig and S1 Table.

## Supporting information

S1 Fig This is the S1 Fig All details figures. (ZIP)

S1 Table This is the S1 Table All details tables. (ZIP)
