# Supplementary figures and images for: Hemodynamic impact of blood viscosity in intracranial atherosclerotic arteries with varying stenosis severity: A non-newtonian computational fluid dynamics patient specific study
Source: PLoS One. 2026 May 28;21(5):e0342713. doi: 10.1371/journal.pone.0342713 (PMC13218500; doi:10.1371/journal.pone.0342713)

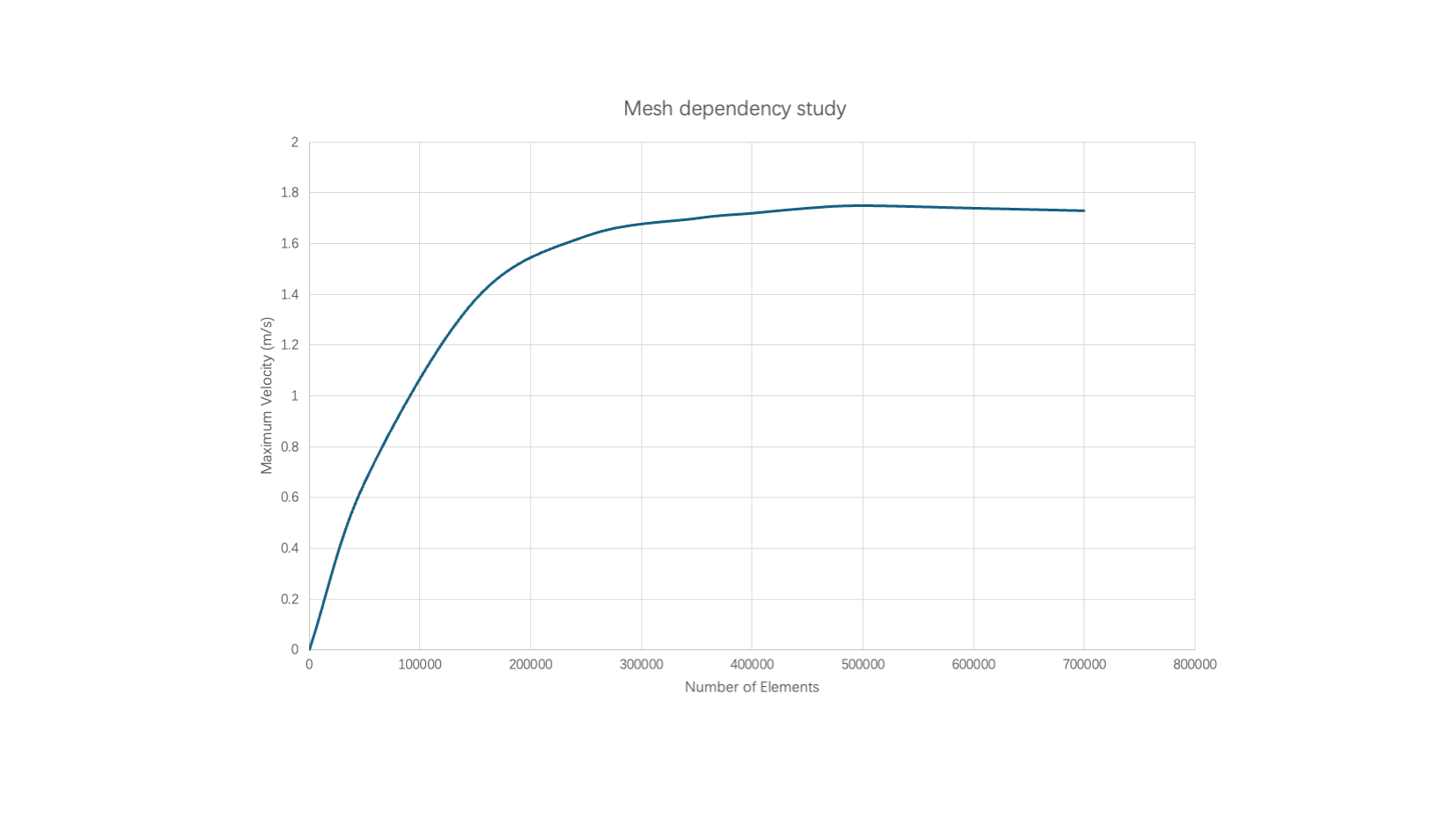

Supplement: S1 Fig — (ZIP) [file pone.0342713.s001.zip › S1 Fig/fig 3.tiff]

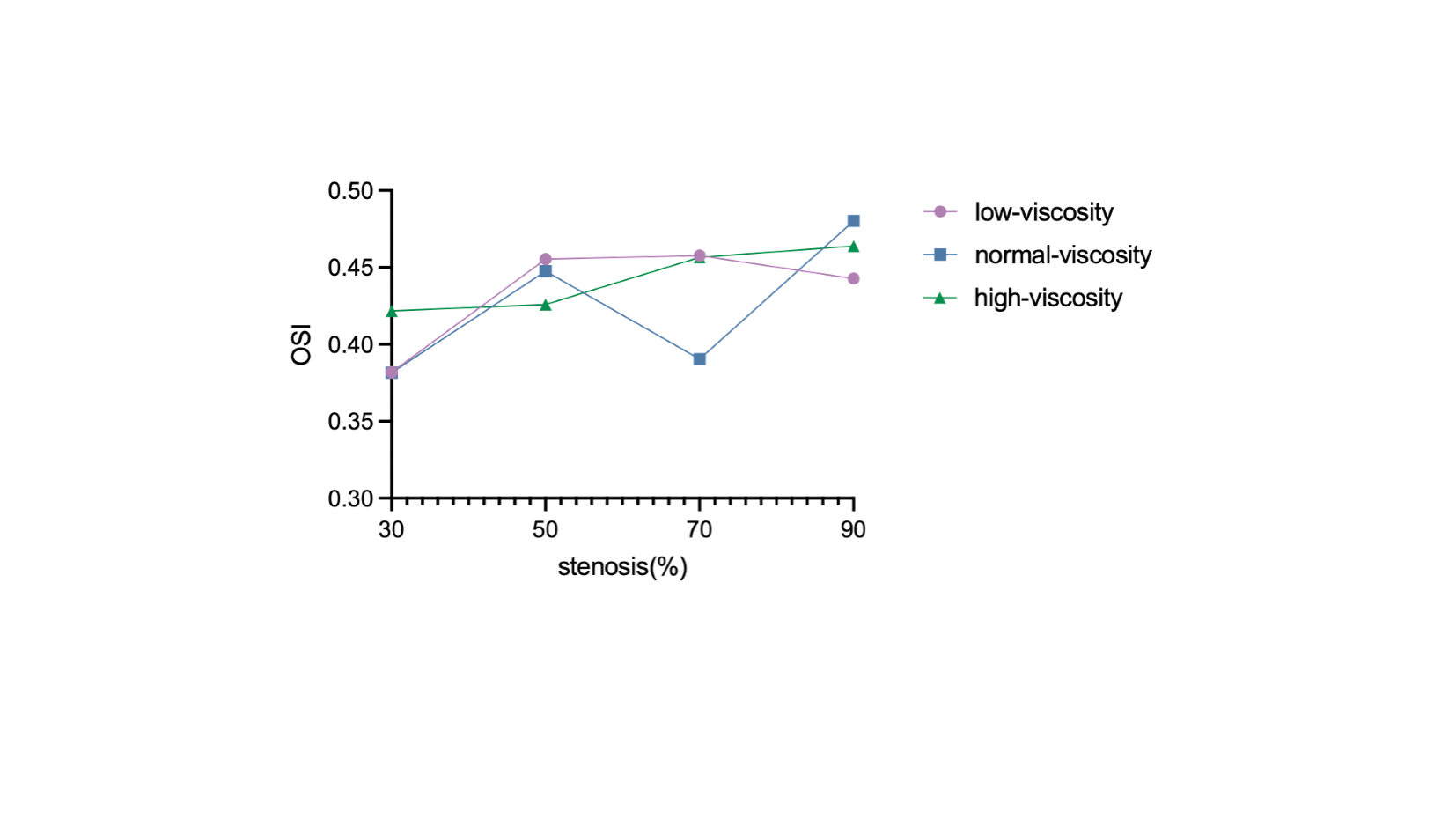

Supplement: S1 Fig — (ZIP) [file pone.0342713.s001.zip › S1 Fig/fig 25.tiff]

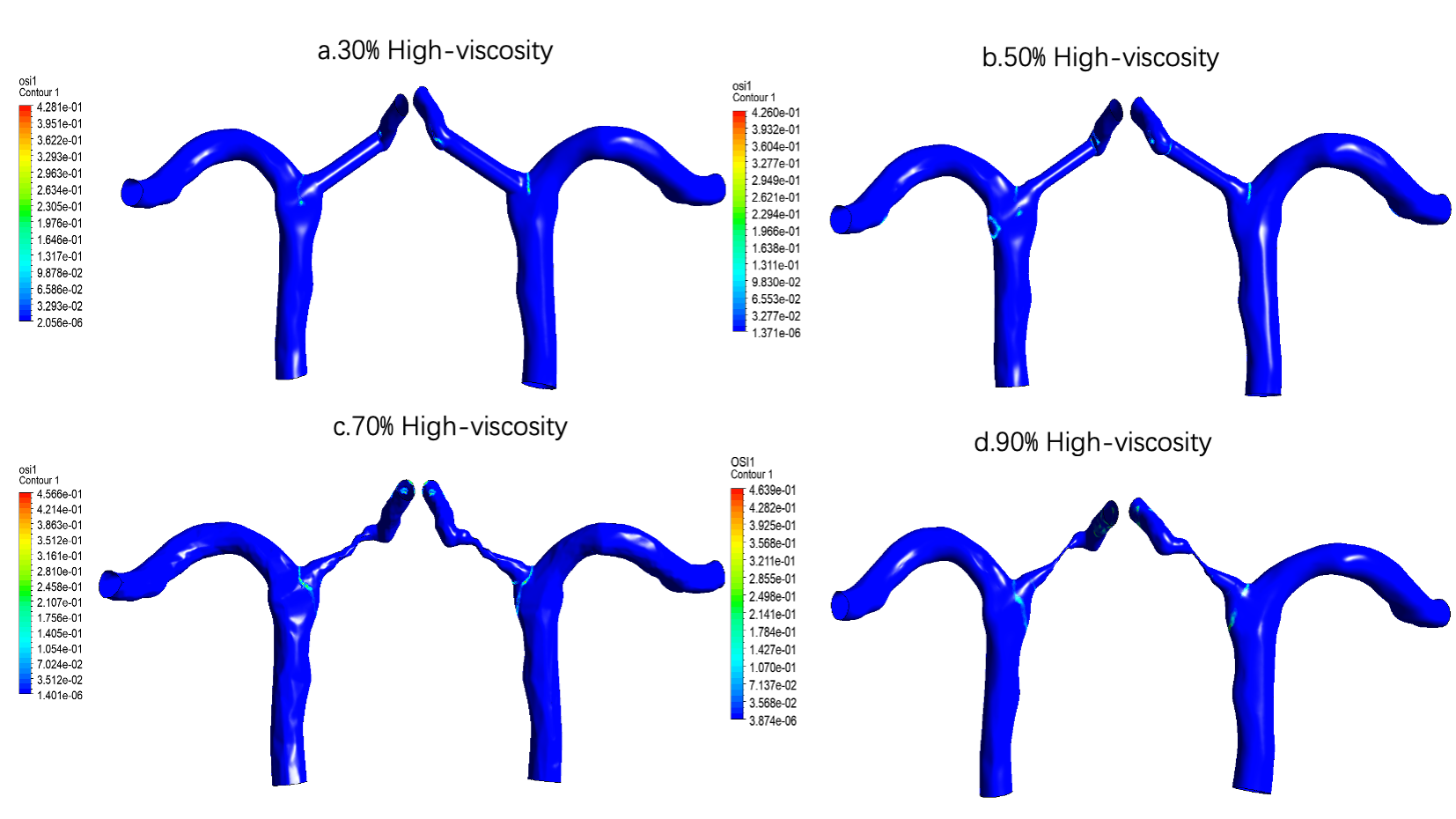

Supplement: S1 Fig — (ZIP) [file pone.0342713.s001.zip › S1 Fig/fig 24.tiff]

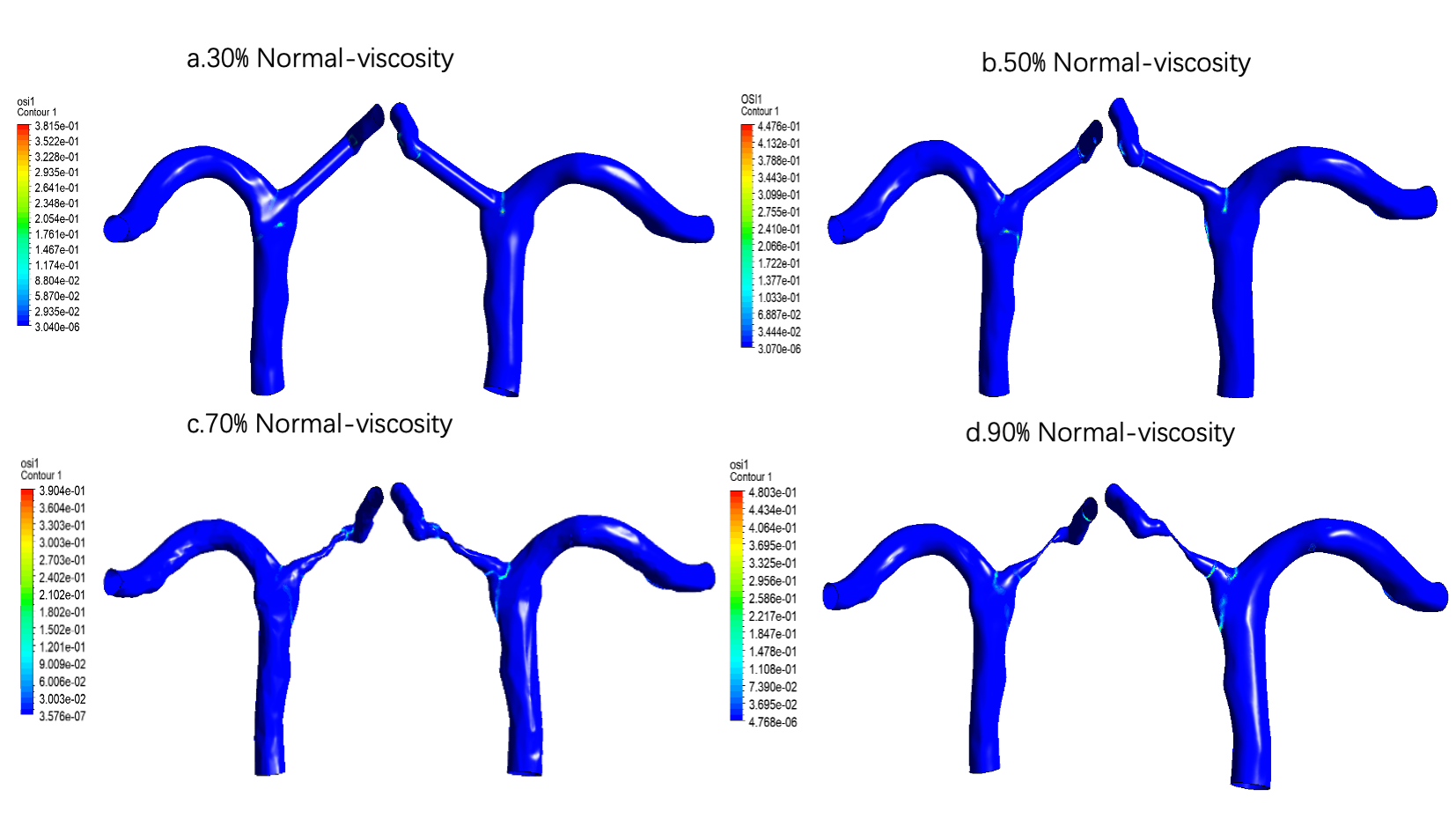

Supplement: S1 Fig — (ZIP) [file pone.0342713.s001.zip › S1 Fig/fig 23.tiff]

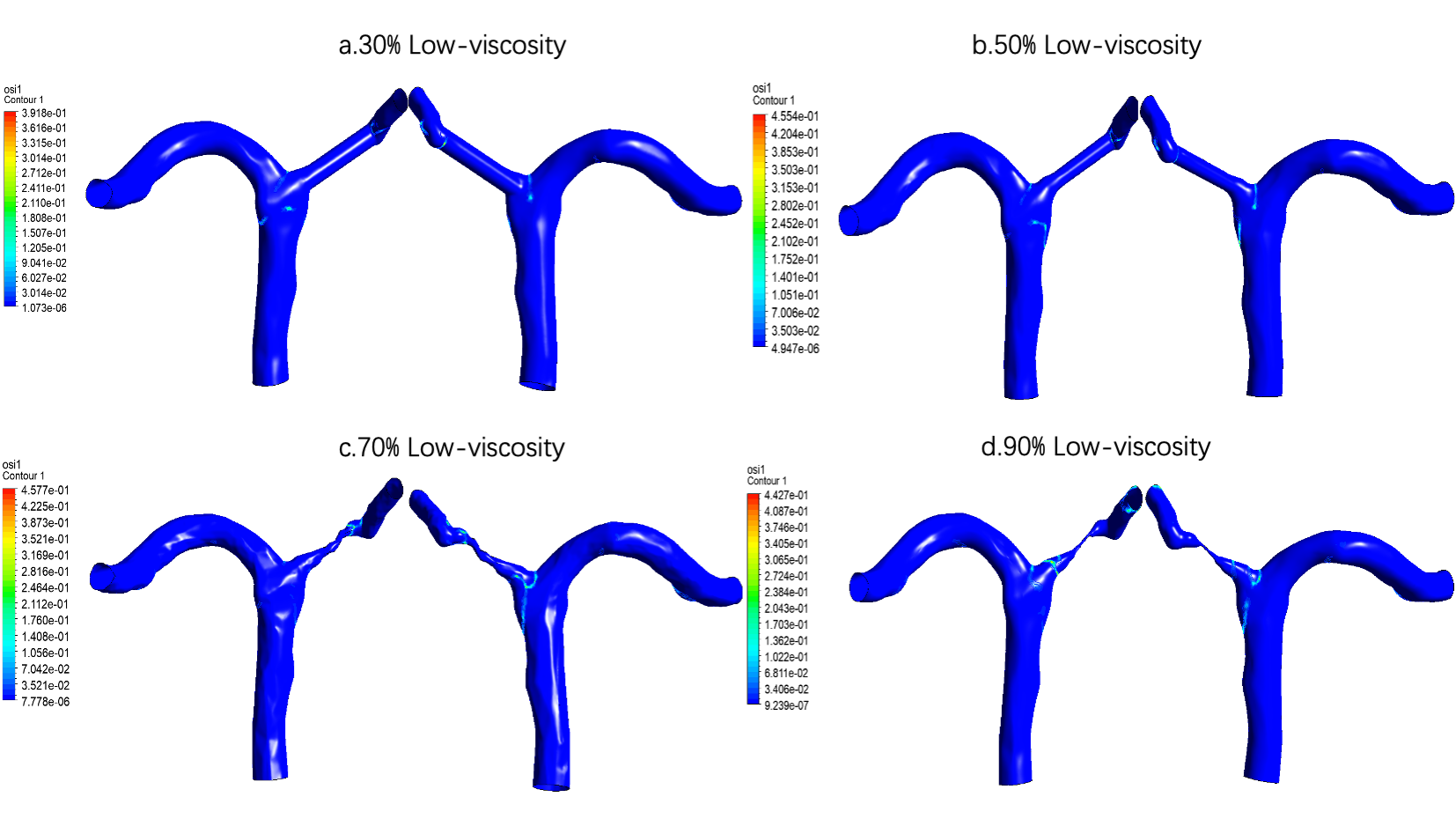

Supplement: S1 Fig — (ZIP) [file pone.0342713.s001.zip › S1 Fig/fig 22.tiff]

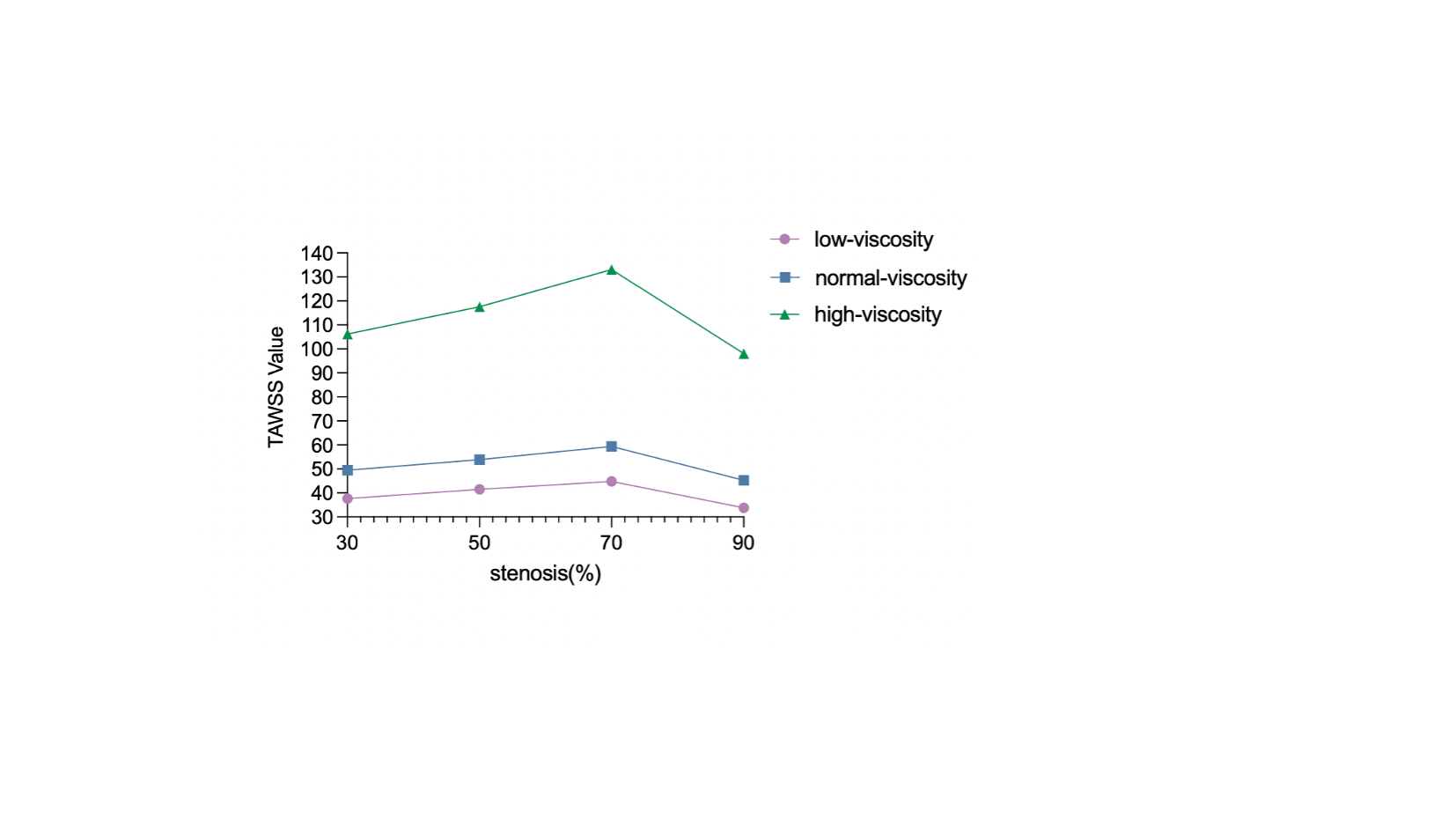

Supplement: S1 Fig — (ZIP) [file pone.0342713.s001.zip › S1 Fig/fig 21.tiff]

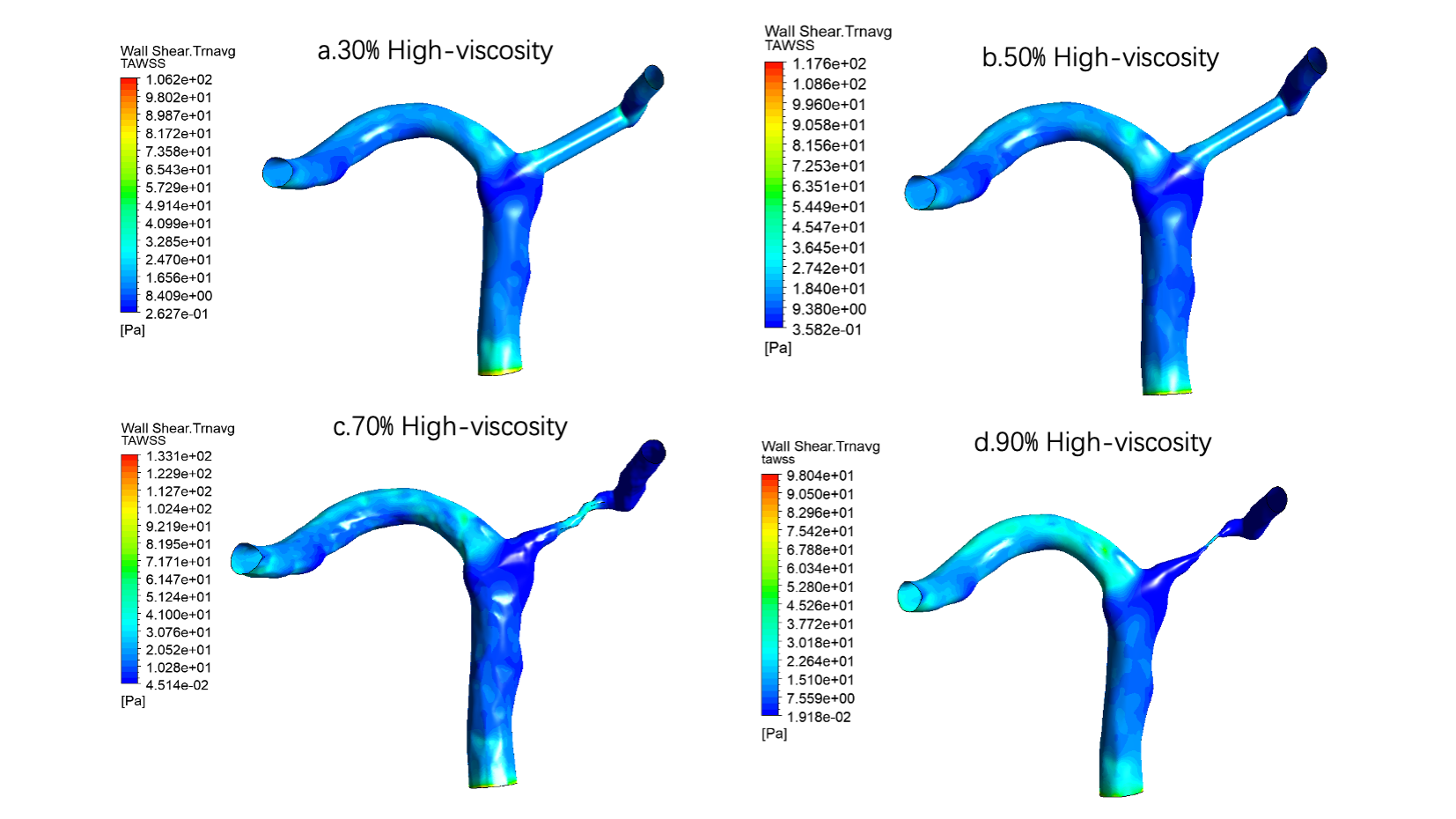

Supplement: S1 Fig — (ZIP) [file pone.0342713.s001.zip › S1 Fig/fig 20.tiff]

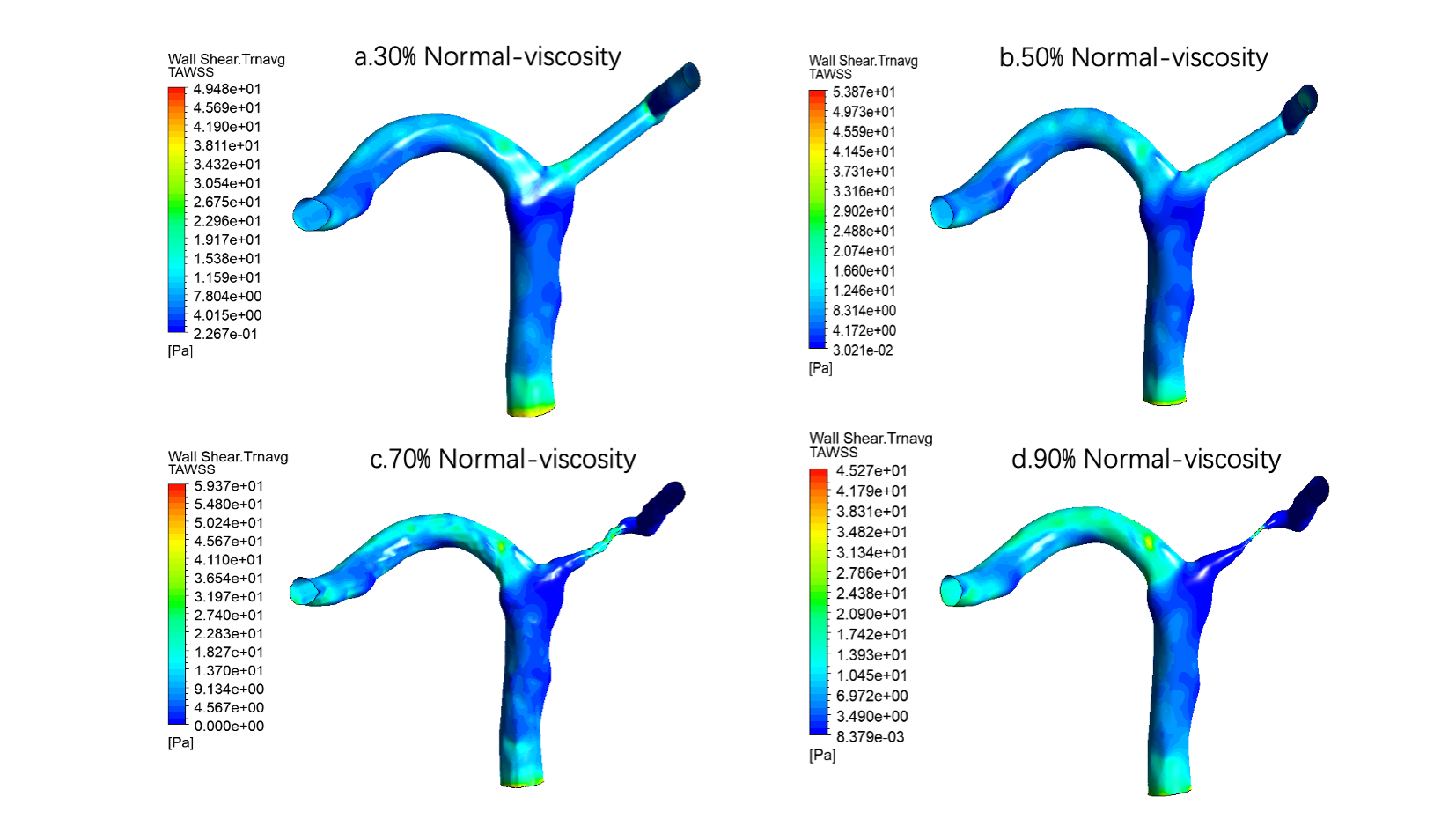

Supplement: S1 Fig — (ZIP) [file pone.0342713.s001.zip › S1 Fig/fig 19.tiff]

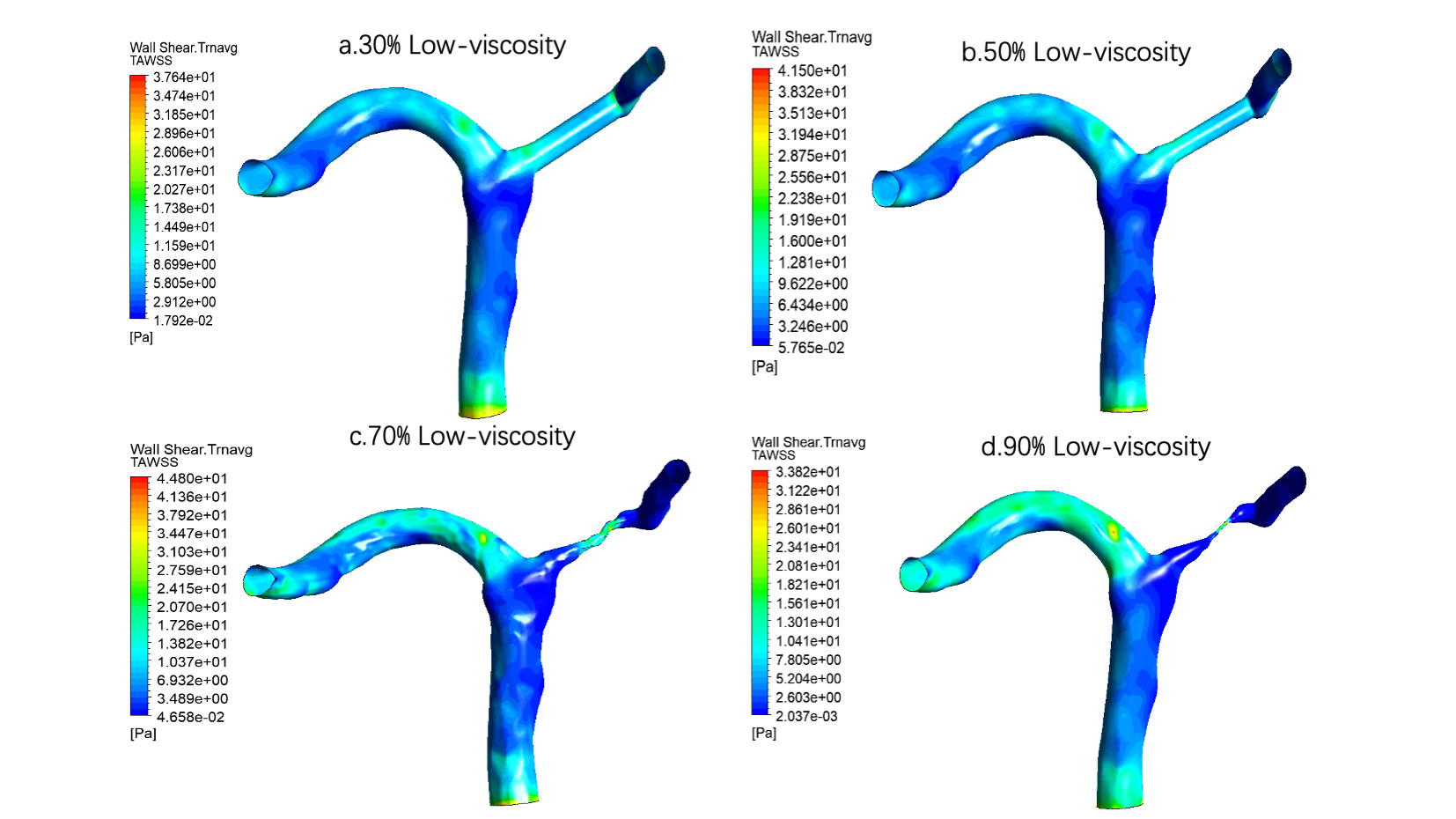

Supplement: S1 Fig — (ZIP) [file pone.0342713.s001.zip › S1 Fig/fig 18.tiff]

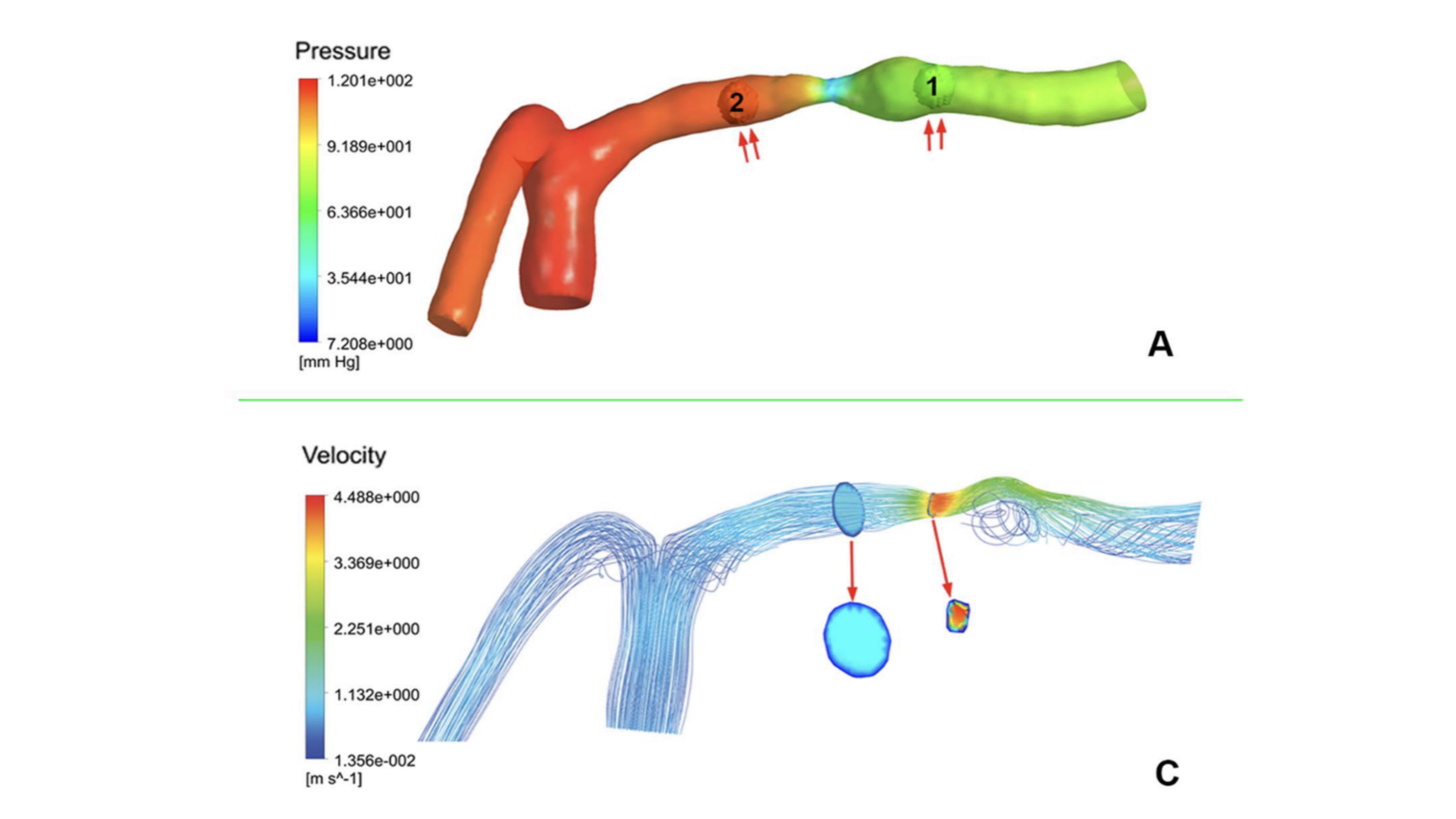

Supplement: S1 Fig — (ZIP) [file pone.0342713.s001.zip › S1 Fig/fig 5.tiff]

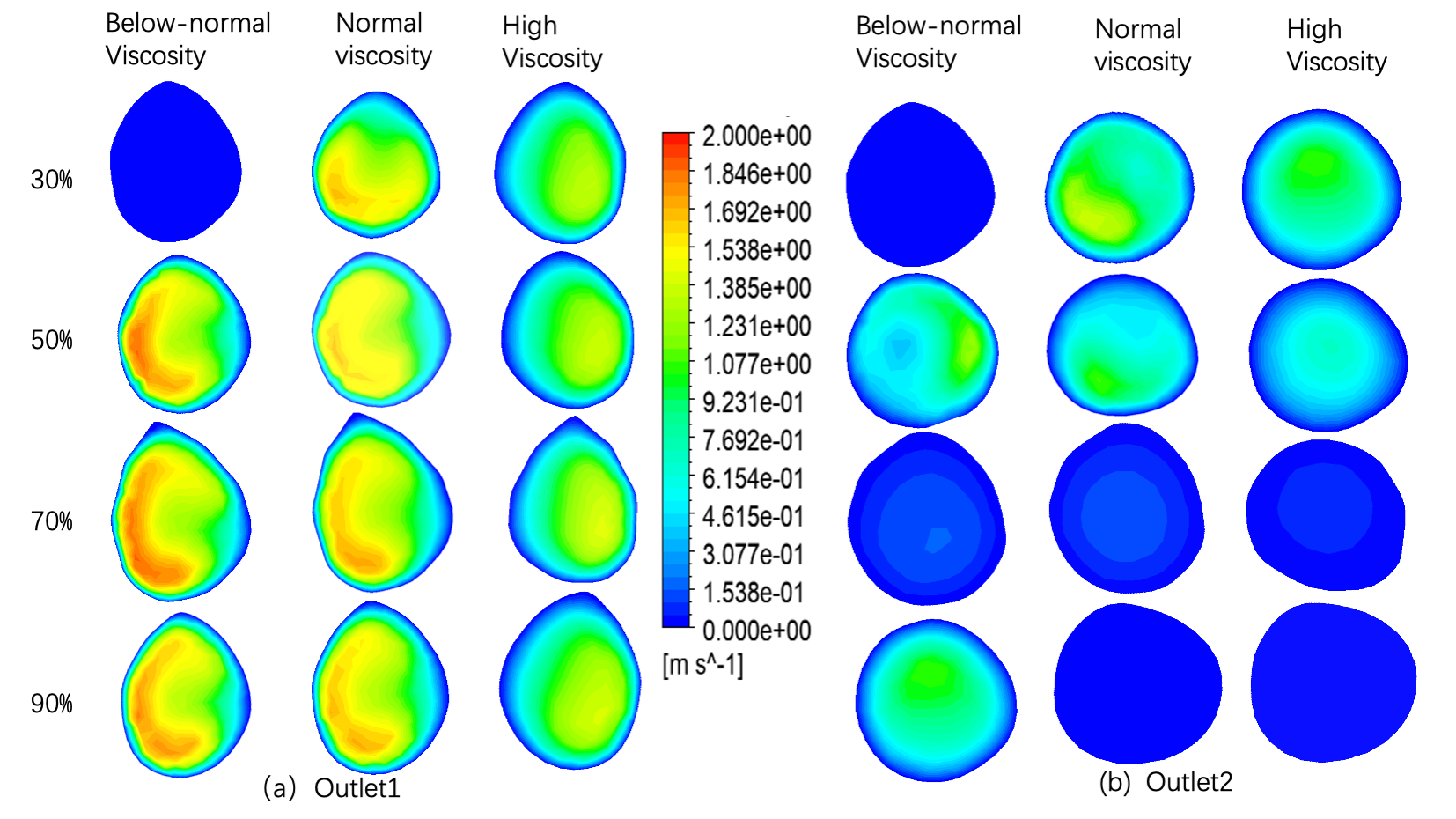

Supplement: S1 Fig — (ZIP) [file pone.0342713.s001.zip › S1 Fig/fig 6.tiff]

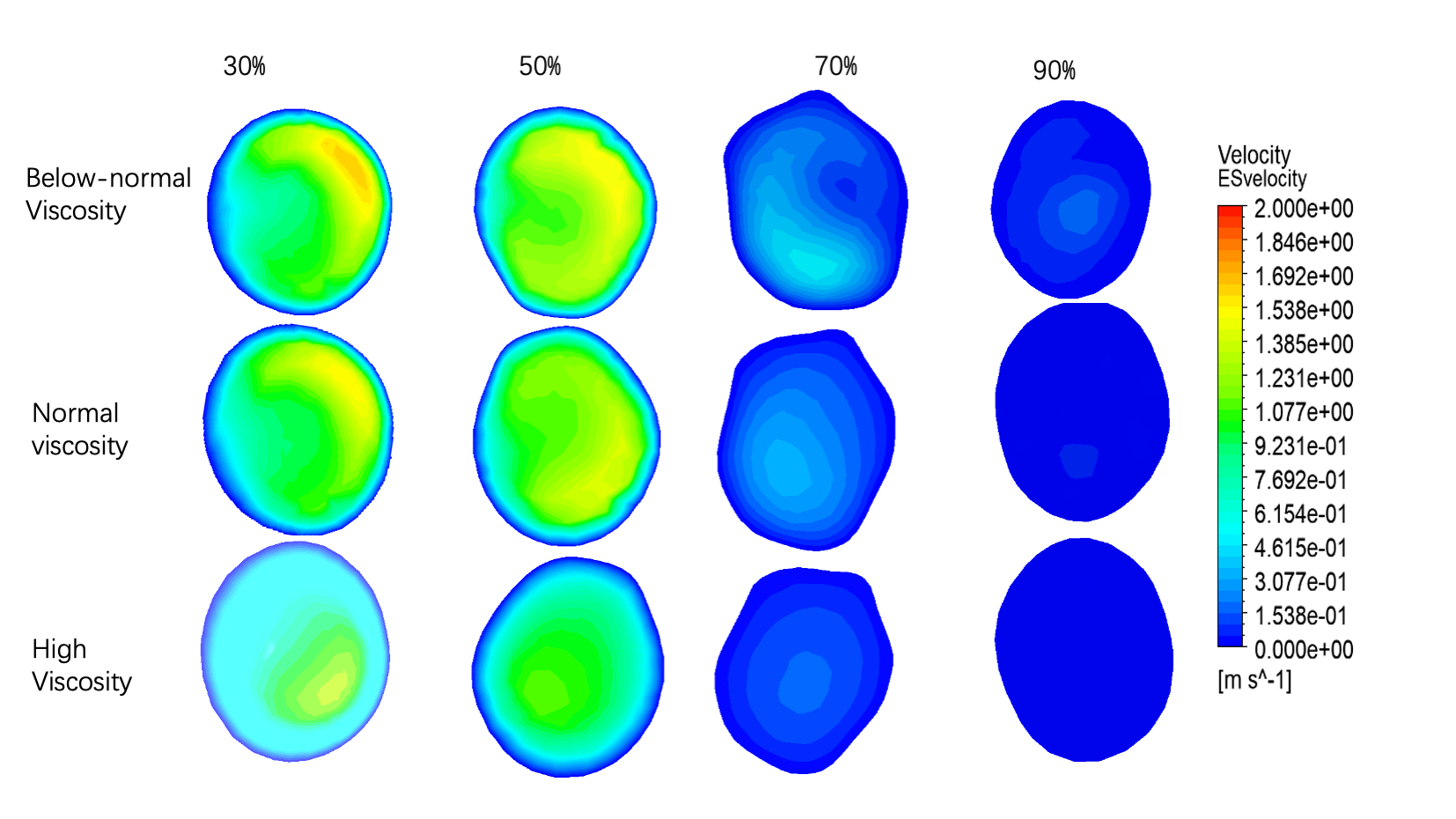

Supplement: S1 Fig — (ZIP) [file pone.0342713.s001.zip › S1 Fig/fig 7.tiff]

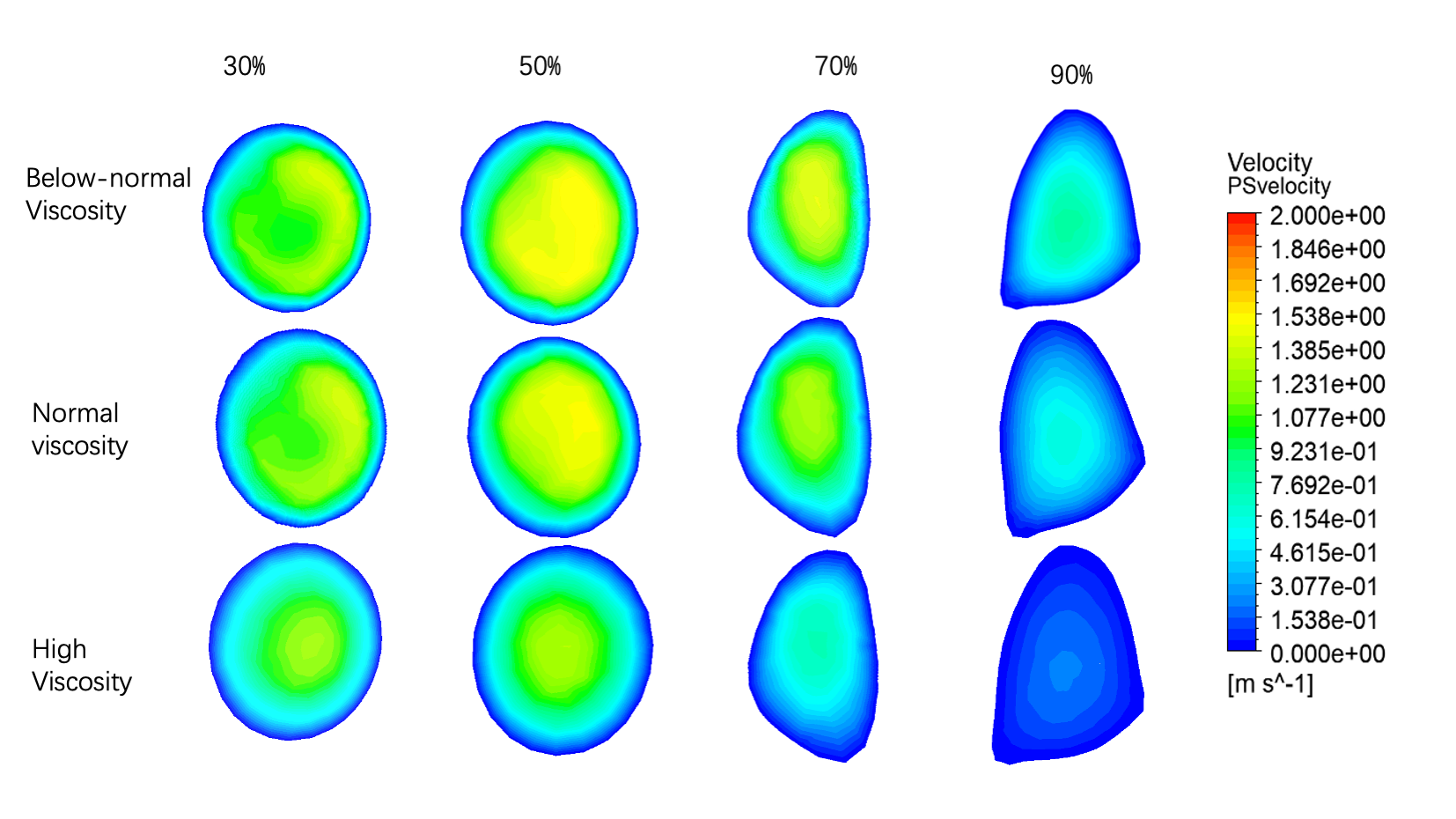

Supplement: S1 Fig — (ZIP) [file pone.0342713.s001.zip › S1 Fig/fig 8 .tiff]

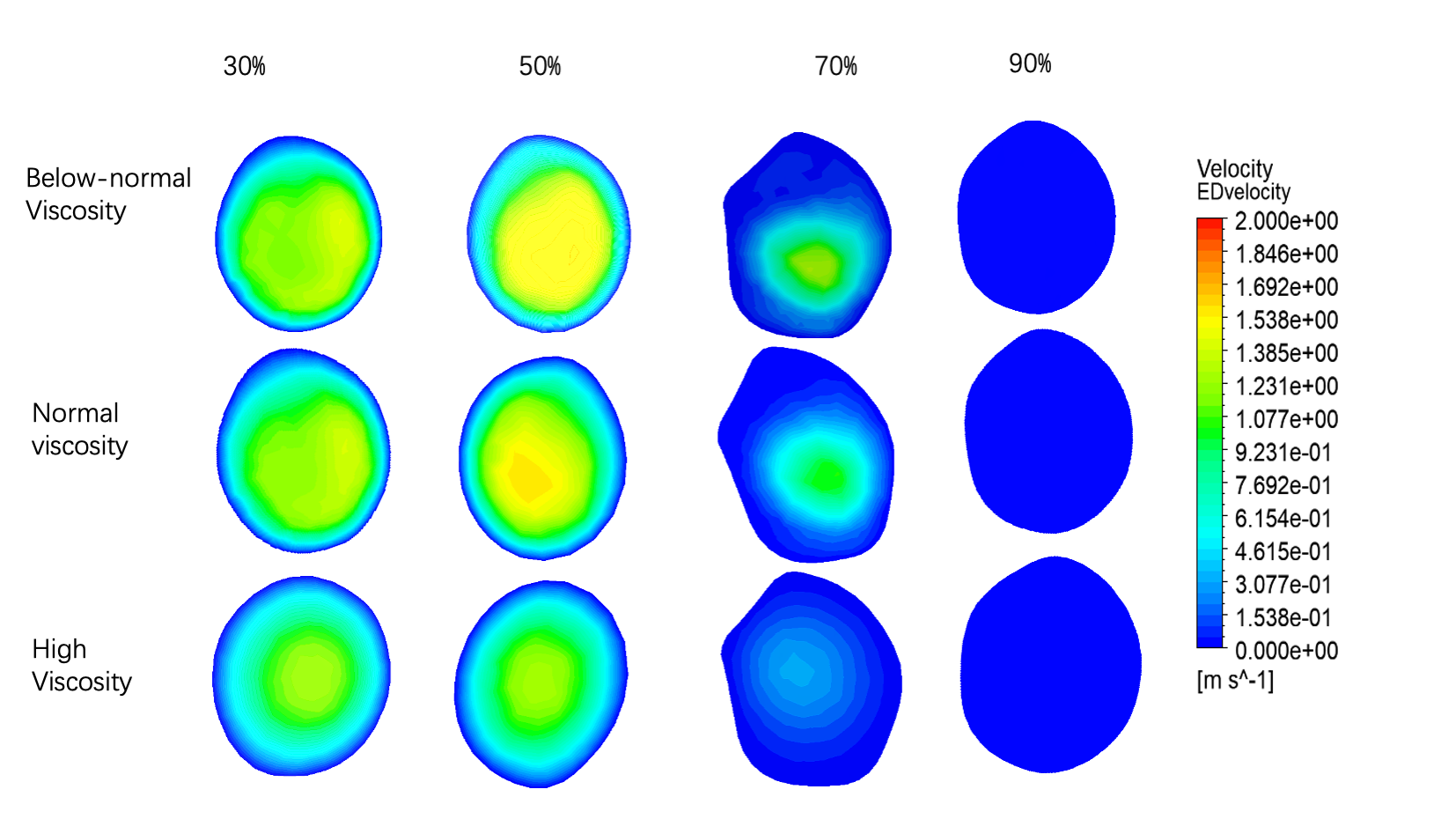

Supplement: S1 Fig — (ZIP) [file pone.0342713.s001.zip › S1 Fig/fig 9.tiff]

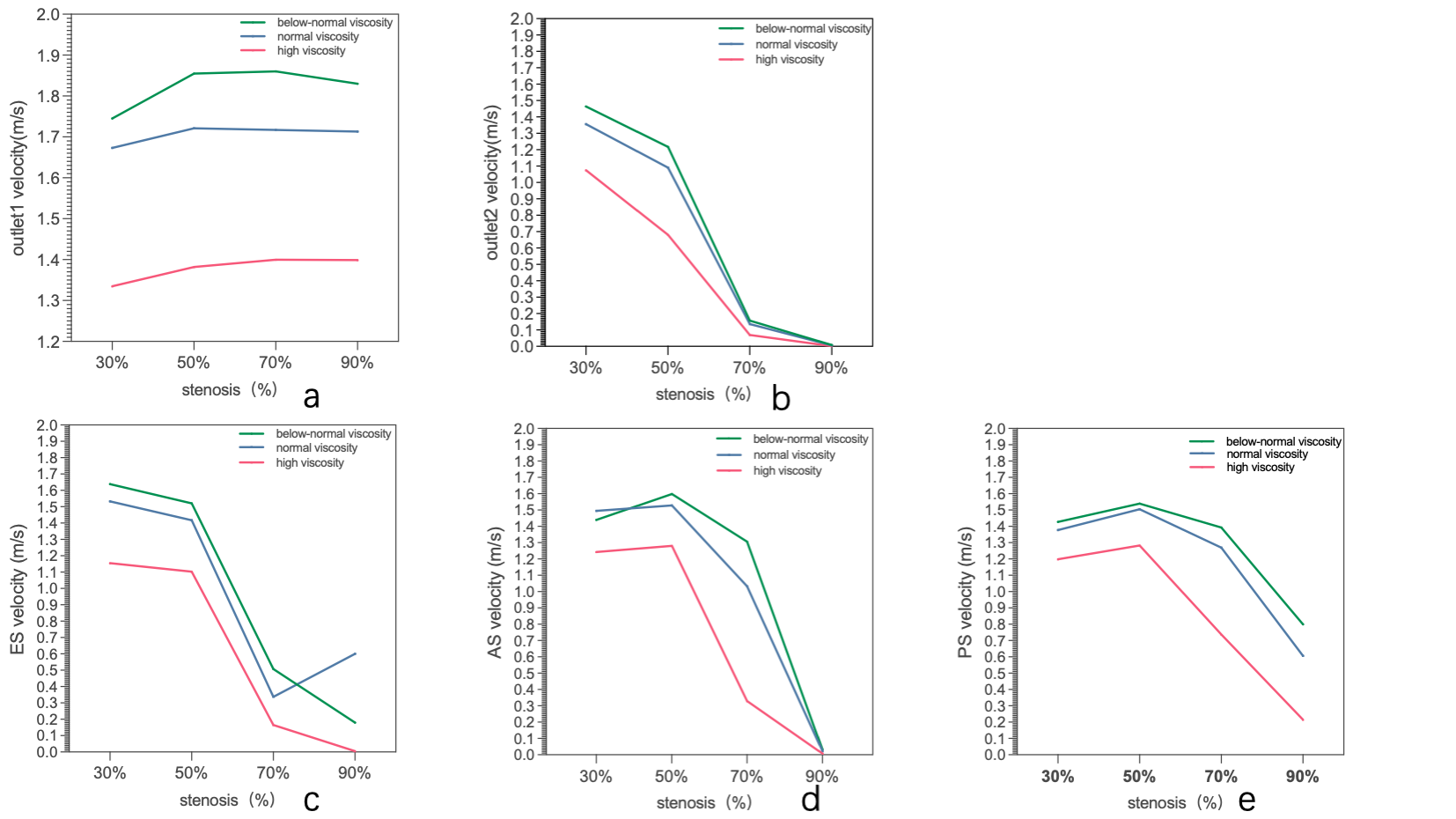

Supplement: S1 Fig — (ZIP) [file pone.0342713.s001.zip › S1 Fig/fig 10.tiff]

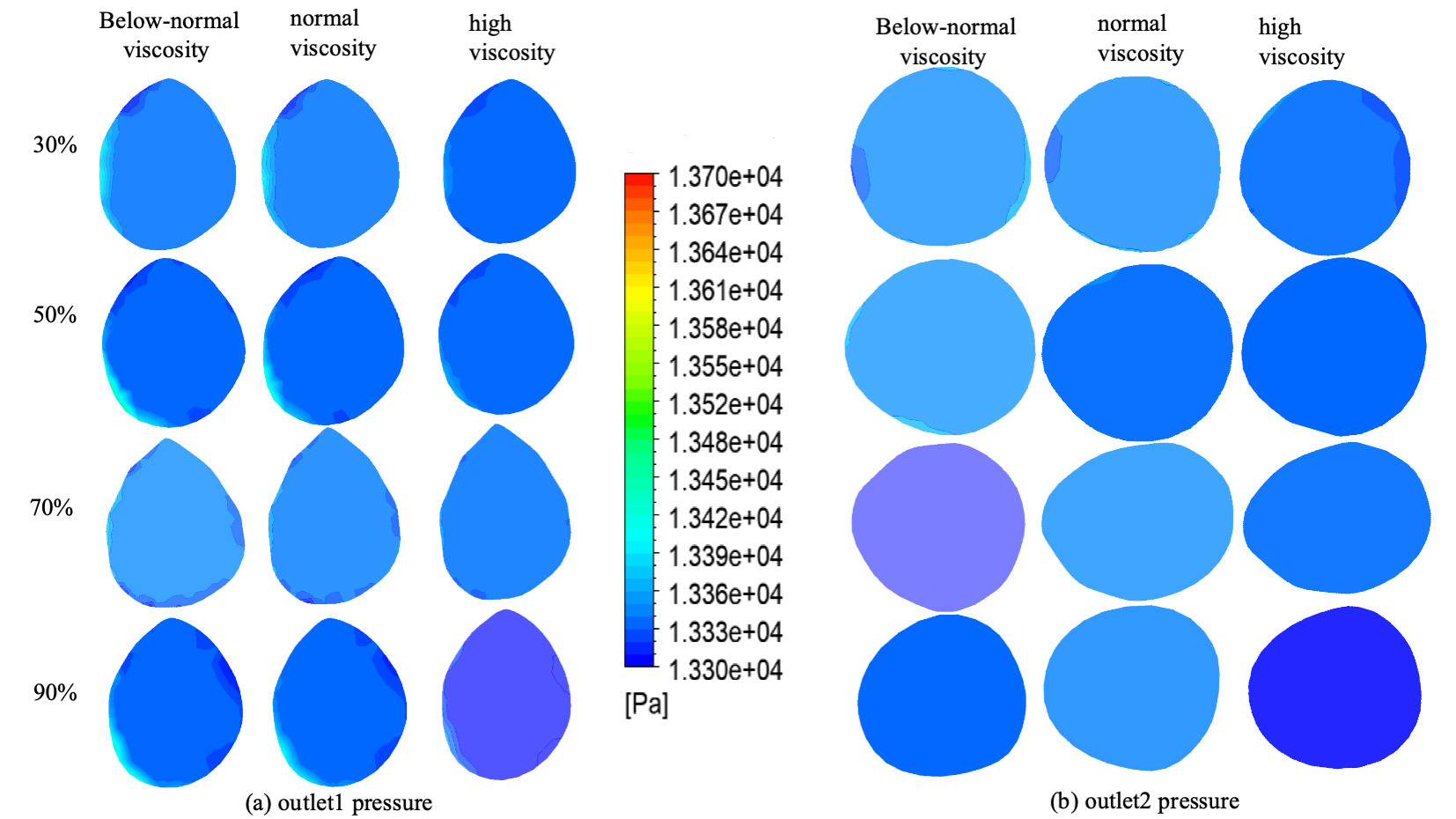

Supplement: S1 Fig — (ZIP) [file pone.0342713.s001.zip › S1 Fig/fig 11.tiff]

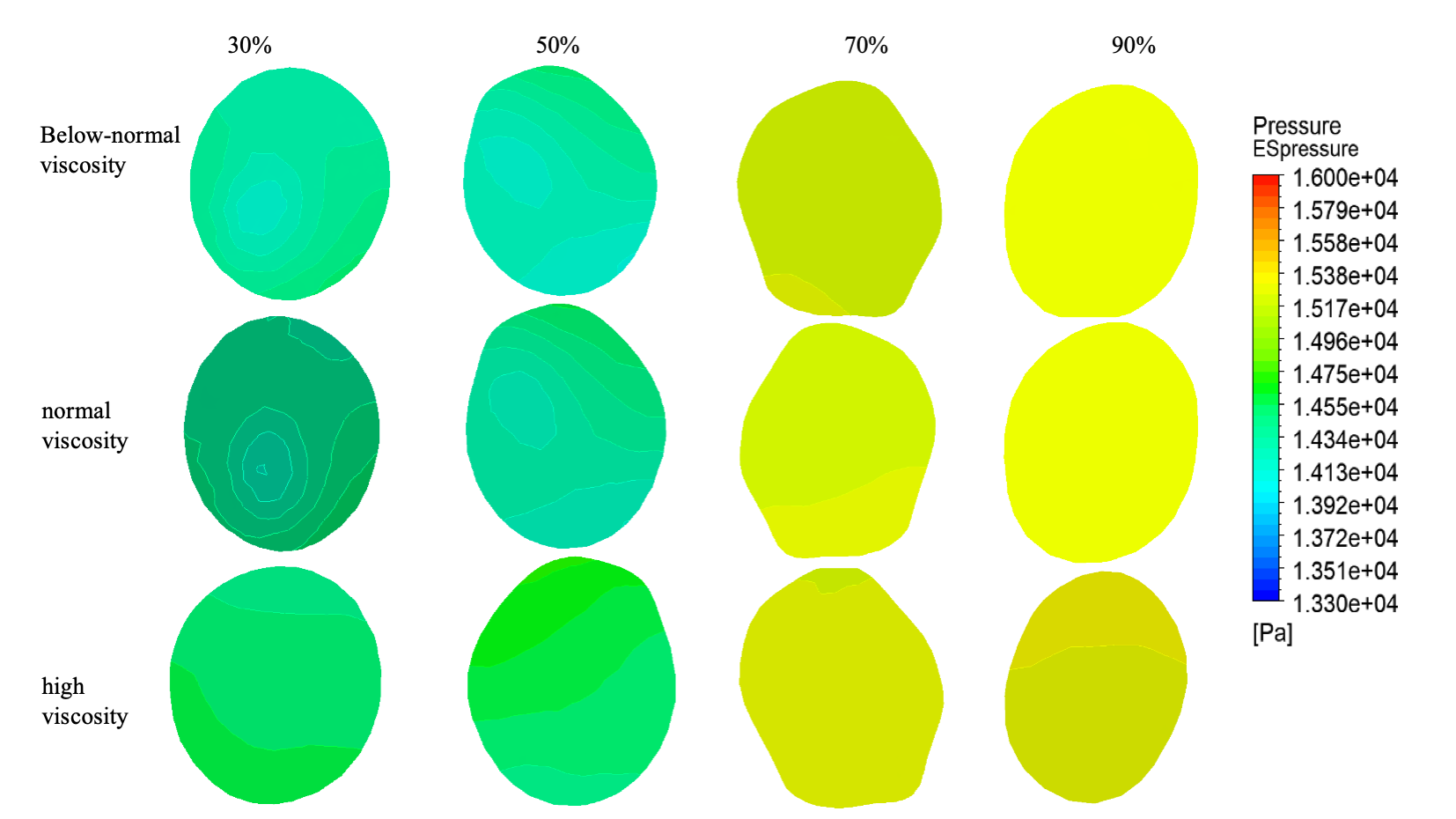

Supplement: S1 Fig — (ZIP) [file pone.0342713.s001.zip › S1 Fig/fig 12.tiff]

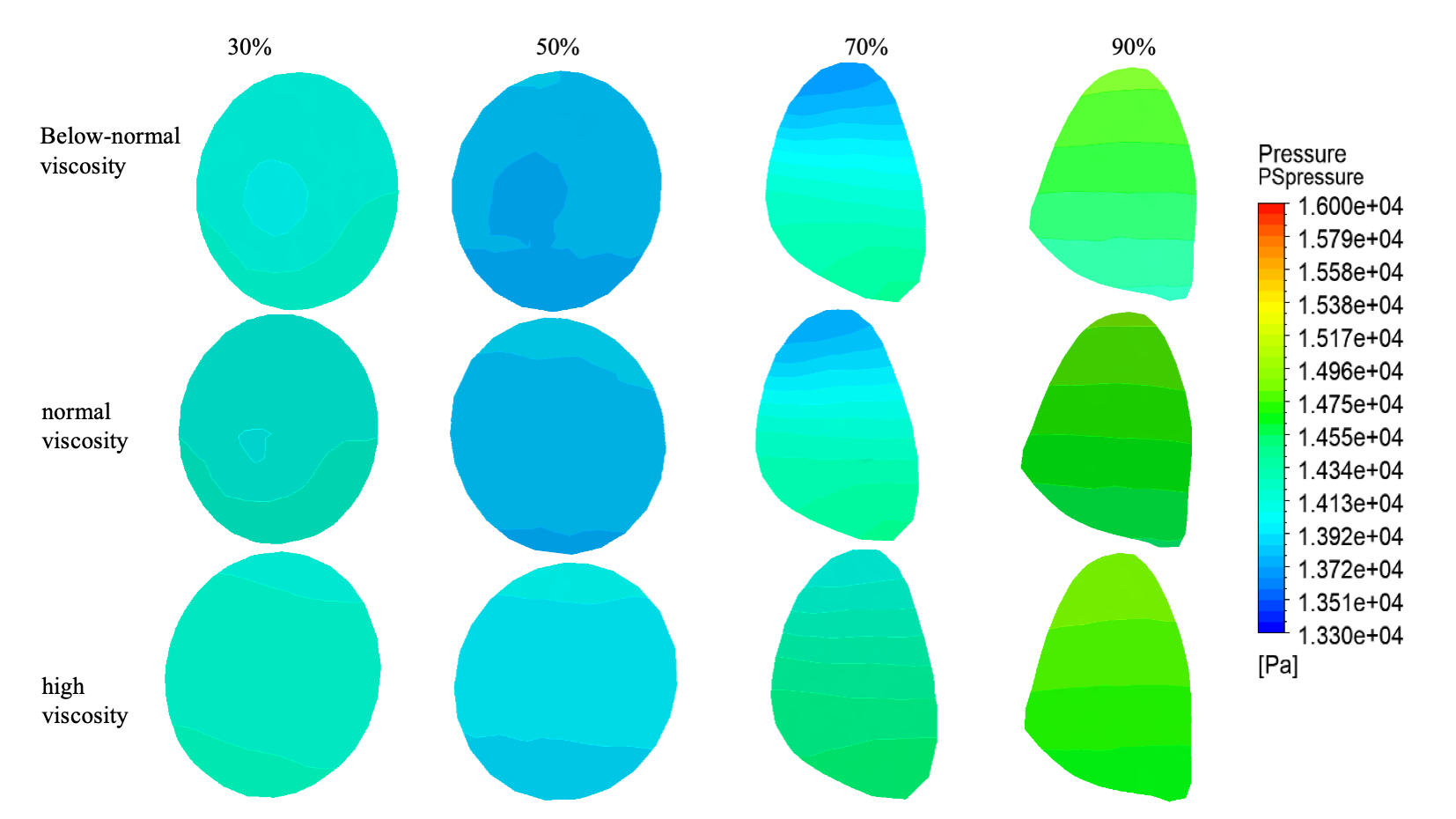

Supplement: S1 Fig — (ZIP) [file pone.0342713.s001.zip › S1 Fig/fig 13.tiff]

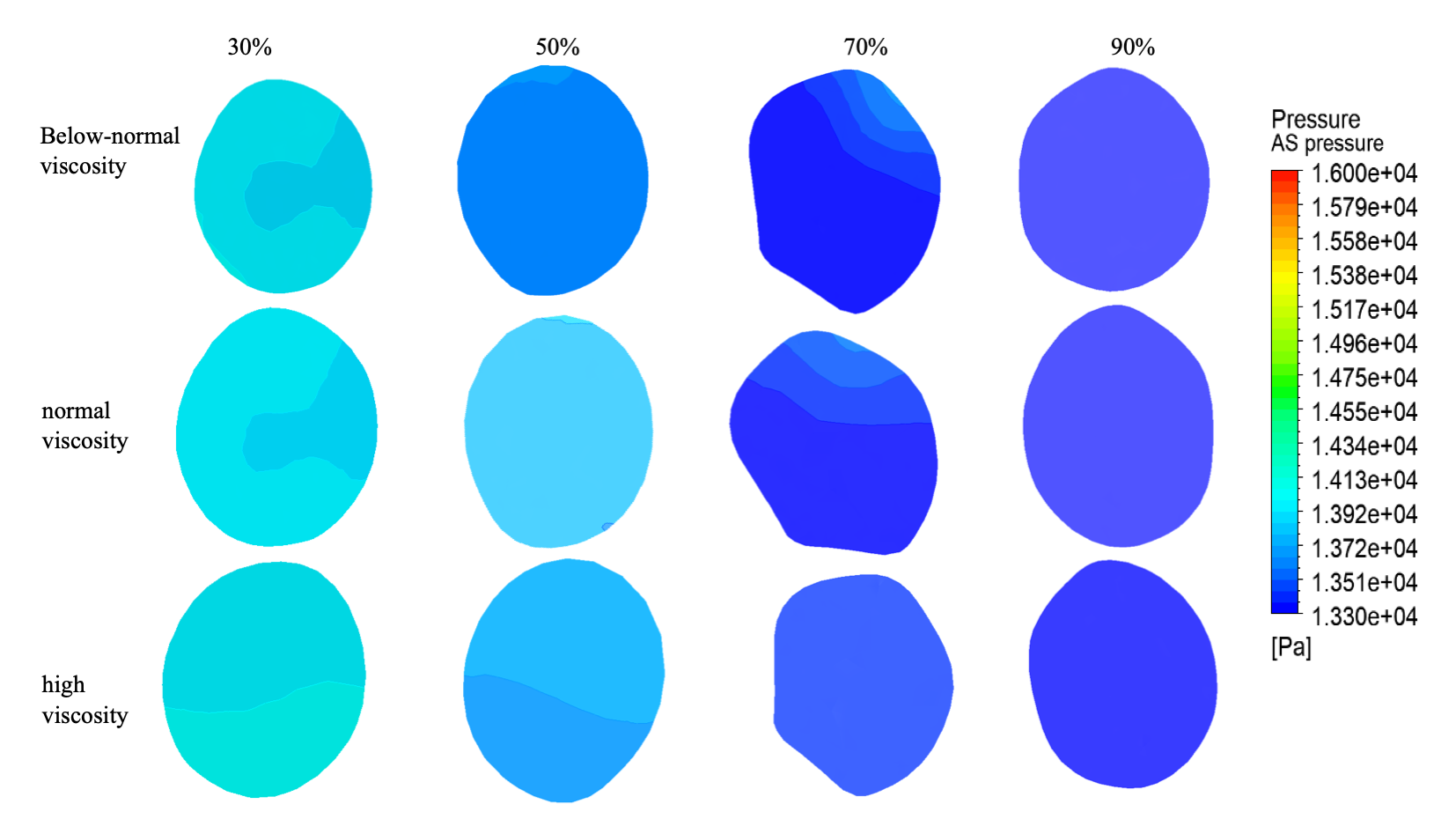

Supplement: S1 Fig — (ZIP) [file pone.0342713.s001.zip › S1 Fig/fig 14.tiff]

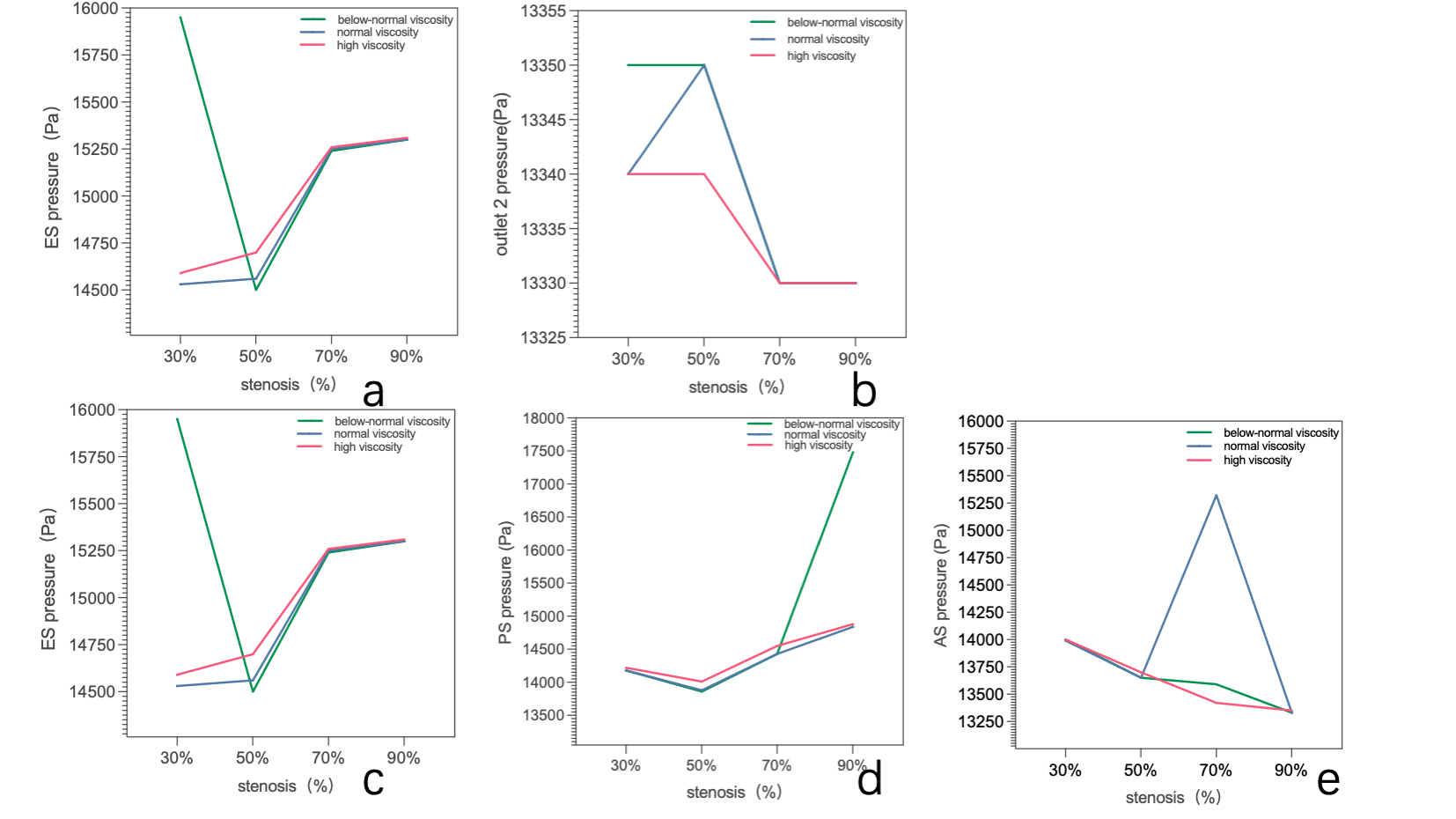

Supplement: S1 Fig — (ZIP) [file pone.0342713.s001.zip › S1 Fig/fig 15.tiff]

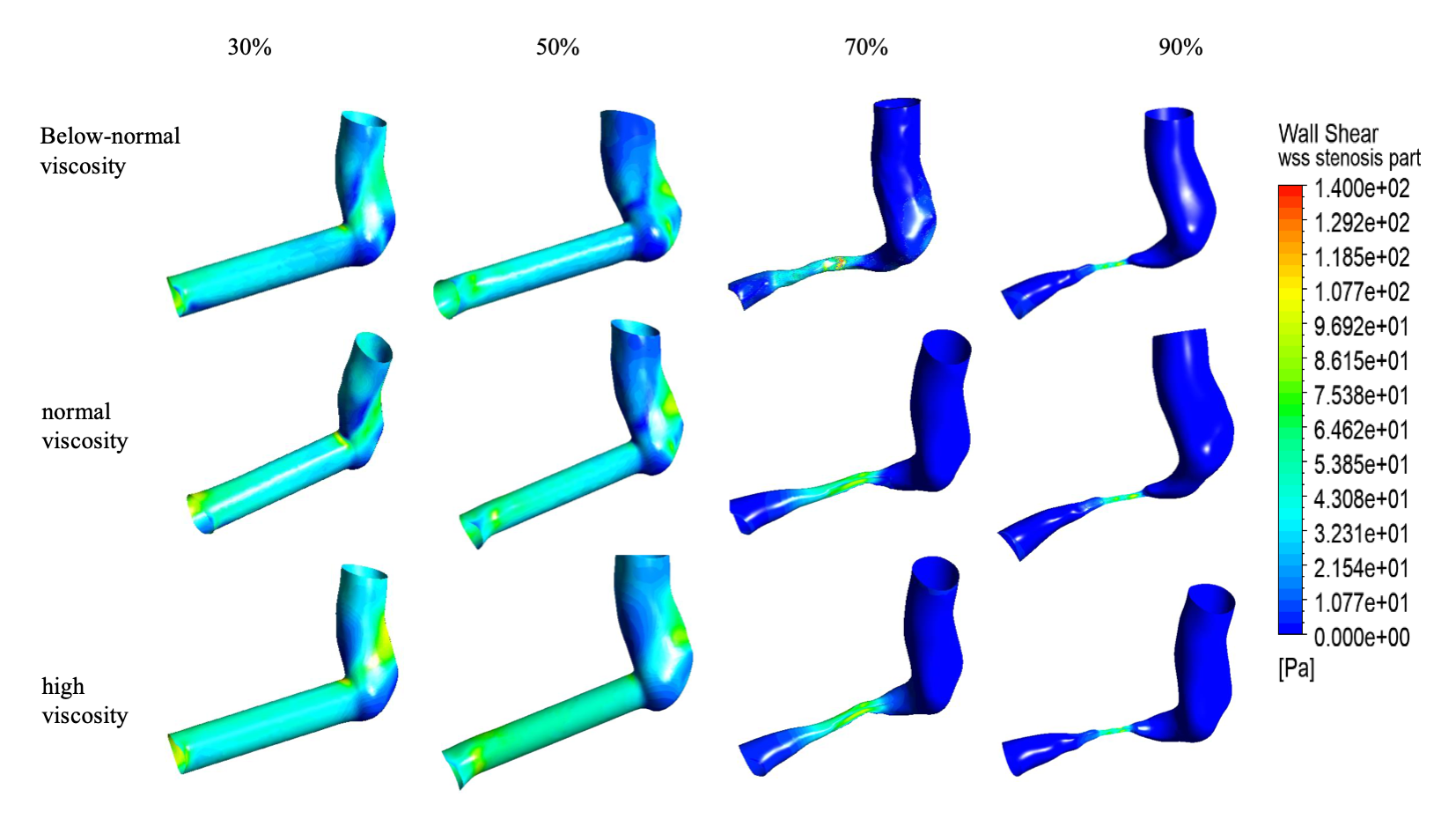

Supplement: S1 Fig — (ZIP) [file pone.0342713.s001.zip › S1 Fig/fig 16.tiff]

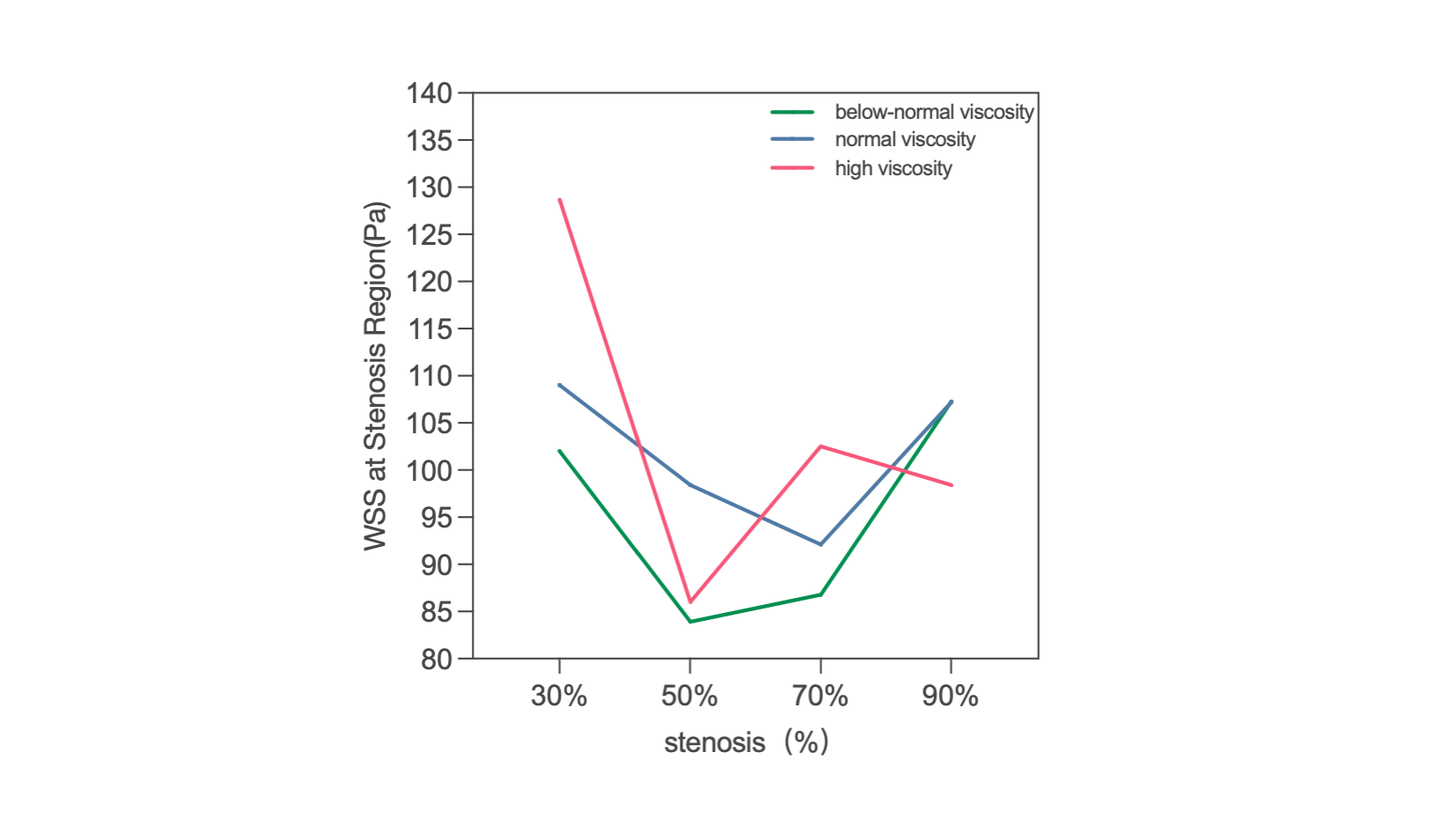

Supplement: S1 Fig — (ZIP) [file pone.0342713.s001.zip › S1 Fig/fig 17.tiff]

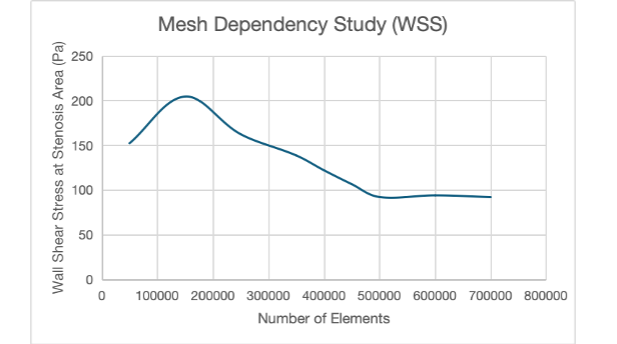

Supplement: S1 Fig — (ZIP) [file pone.0342713.s001.zip › S1 Fig/fig 4.tiff]

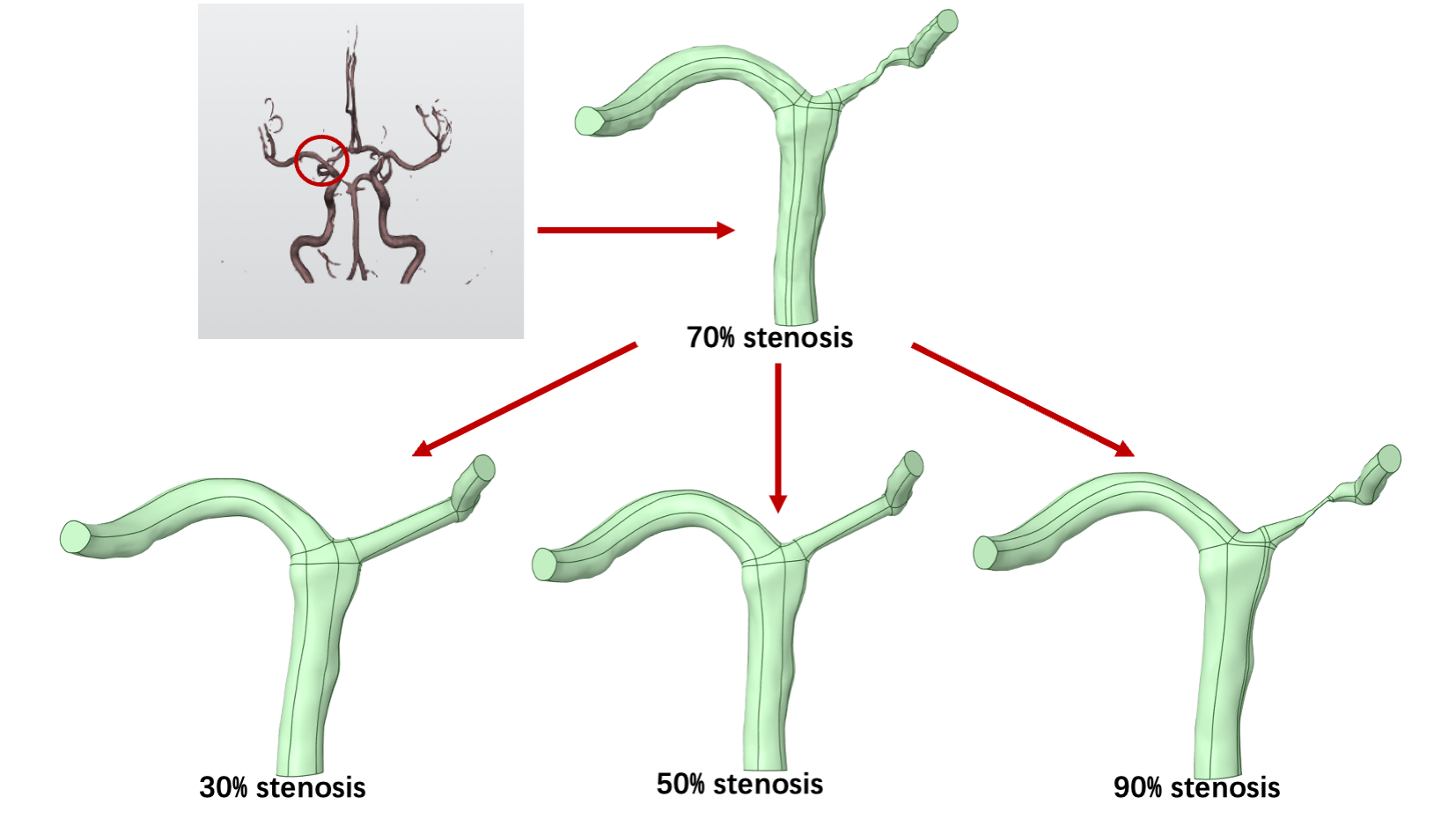

Supplement: S1 Fig — (ZIP) [file pone.0342713.s001.zip › S1 Fig/fig 1 .tiff]

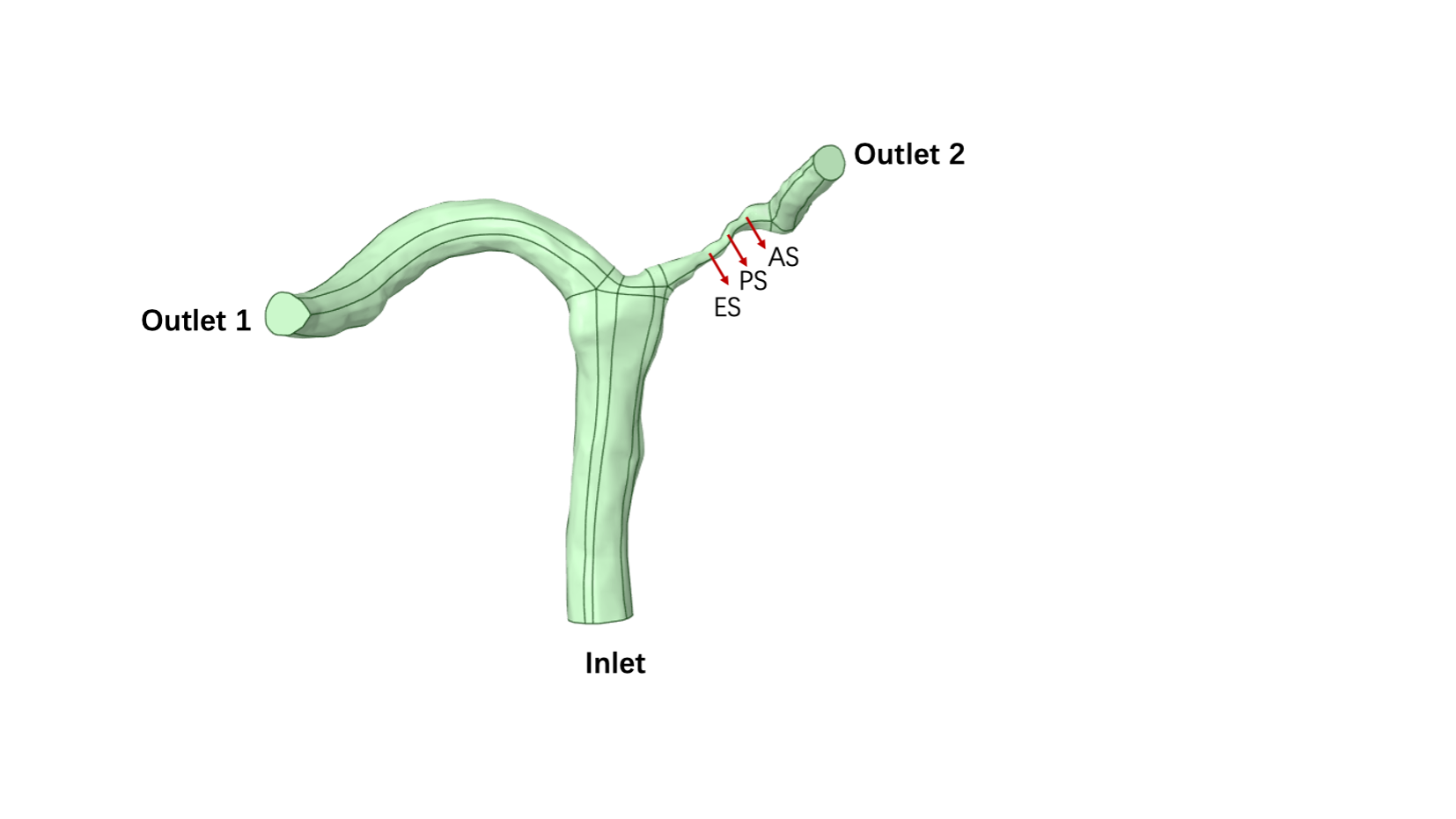

Supplement: S1 Fig — (ZIP) [file pone.0342713.s001.zip › S1 Fig/fig 2.tiff]
