# Supplementary material for: Hemodynamic impact of blood viscosity in intracranial atherosclerotic arteries with varying stenosis severity: A non-newtonian computational fluid dynamics patient specific study
Source: PLoS One. 2026 May 28;21(5):e0342713. doi: 10.1371/journal.pone.0342713 (PMC13218500; doi:10.1371/journal.pone.0342713)
Supplement: S1 Table — (ZIP) [file pone.0342713.s002.zip › S1 Table/S1 Table.docx]

**Table 1. Model parameters, boundary conditions, and numerical setup used in the simulations.**

| **Category** | **Parameter** | **Value** | **reference** |
| --- | --- | --- | --- |
| **Blood density** | $\rho$ | 1060kg·m^-3^ | [21] |
| **Rheology model** | Carreau model | - |  |
| **low-viscosity** | \|  \| \| --- \|  \| Power-law index \| \| --- \|   （n） | 0.33 | [11] |
|  | Time constant($\lambda$) | 12.448s | [11] |
|  | Zero-shear viscosity($\mu0$) | 0.0178 Pa·s | [11] |
|  | Infinite-shear viscosity($\mu\infty$) | 0.00257 Pa·s | [11] |
| **Normal-viscosity** | \|  \| \| --- \|  \| Power-law index \| \| --- \|   (n) | 0.3568 | [11] |
|  | Time constant($\lambda$) | 3.313s | [11] |
|  | Zero-shear viscosity($\mu0$) | 0.056 Pa·s | [11] |
|  | Infinite-shear viscosity($\mu\infty$) | 0.0035 Pa·s | [11] |
| **High-viscosity** | \|  \| \| --- \|  \| Power-law index \| \| --- \|   (n) | 0.39 | [11] |
|  | Time constant($\lambda$) | 103.09s | [11] |
|  | Zero-shear viscosity($\mu0$) | 0.8592 Pa·s | [11] |
|  | Infinite-shear viscosity($\mu\infty$) | 0.00802 Pa·s | [11] |
| **Boundary condition** | Inlet (velocity-inlet) | Pulsatile velocity waveform implemented via UDF | [51] |
|  | Outlet1&2  (pressure-outlet) | Flow-dependent resistance-based outlet condition implemented via UDF | [51] |
| **Numerical setup** | Solver type | transient |  |
|  | Flow model | Laminar |  |
|  | Residual threshold | 1×10^−5^ |  |
| **Transient setting** | Cardiac cycle | 1s |  |
|  | Time-step size | 0.002s |  |
|  | Number of time steps | 500 |  |

Table 2. Quantitative comparison of key hemodynamic parameters between the present simulations and the clinically grounded CFD study reported by Leng et al. (2014).

|  | Reference study | Present study | Relative difference (%) |
| --- | --- | --- | --- |
| Pressure ratio | 0.790 | 0.876 | 10.95% |
| Velocity ratio | 5.2 | 5.06 | 2.69% |

**Table 3** **Summary of computed hemodynamic parameters under different stenosis severities (30%, 50%, 70%, and 90%) and blood viscosity conditions (below-normal, normal, and high).**

|  |  | Below normal viscosity | | | | Normal viscosity | | | | High viscosity | | | | |
| --- | --- | --- | --- | --- | --- | --- | --- | --- | --- | --- | --- | --- | --- | --- |
|  |  | 30% | 50% | 70% | 90% | 30% | 50% | 70% | 90% | 30% | 50% | 70% | 90% |  |
| Outlet1 | Pressure | 13390 | 13450 | 13360 | 13430 | 13390 | 13430 | 13350 | 13410 | 13360 | 13370 | 13350 | 13660 |  |
|  | Velocity | 1.745 | 1.855 | 1.86 | 1.83 | 1.673 | 1.721 | 1.717 | 1.713 | 1.335 | 1.382 | 1.4 | 1.399 |  |
| Outlet2 | Pressure | 13350 | 13350 | 13330 | 13330 | 13340 | 13350 | 13330 | 13330 | 13340 | 13340 | 13330 | 13330 |  |
|  | Velocity | 1.464 | 1.217 | 0.1567 | 0.008 | 1.356 | 1.09 | 0.1358 | 0.0056 | 1.075 | 0.6811 | 0.06942 | 0.002 |  |
| ES | Pressure | 15950 | 14500 | 15240 | 15300 | 14530 | 14560 | 15250 | 15300 | 14590 | 14700 | 15260 | 15310 |  |
|  | Velocity | 1.638 | 1.52 | 0.5076 | 0.179 | 1.533 | 1.417 | 0.3374 | 0.05641 | 1.155 | 1.103 | 0.1654 | 0.005 |  |
| PS | Pressure | 14180 | 13860 | 14430 | 17480 | 14180 | 13880 | 14430 | 14840 | 14220 | 14010 | 14550 | 14880 |  |
|  | Velocity | 1.427 | 1.539 | 1.393 | 0.7995 | 1.377 | 1.505 | 1.269 | 0.6064 | 1.198 | 1.283 | 0.7377 | 0.2148 |  |
| AS | Pressure | 14000 | 13650 | 13590 | 13330 | 13990 | 13650 | 15320 | 13330 | 14000 | 13700 | 13420 | 13350 |  |
|  | Velocity | 1.439 | 1.598 | 1.306 | 0.03143 | 1.495 | 1.528 | 1.033 | 0.02142 | 1.243 | 1.281 | 0.329 | 0.007 |  |
| Stenosis part | WSS | 102 | 83.93 | 86.79 | 107.3 | 109 | 98.41 | 92.12 | 107.2 | 128.7 | 86.01 | 102.5 | 98.41 |  |

**Table 4．TAWSS denotes time-averaged wall shear stress, defined as the mean magnitude of wall shear stress over one cardiac cycle. All values were obtained from transient simulations using the final cardiac cycle to ensure periodic stability. Different viscosity conditions (below-normal, normal, and high) represent inter-individual variability in blood rheology.**

| **Stenosis(%)** | **Low-viscosity TAWSS（Pa）** | **Normal-viscosity**  **TAWSS(Pa)** | **High-viscosity**  **TAWSS(Pa)** |
| --- | --- | --- | --- |
| **30** | 37.64 | 49.48 | 106.2 |
| **50** | 41.50 | 53.87 | 117.6 |
| **70** | 44.80 | 59.37 | 133.1 |
| **90** | 33.82 | 45.27 | 98.04 |

**Table 5. OSI denotes oscillatory shear index, which quantifies the directional oscillation of wall shear stress over a cardiac cycle. OSI ranges from 0 (unidirectional flow) to 0.5 (fully oscillatory flow). All values were obtained from transient simulations using the final cardiac cycle to ensure periodic stability. Different viscosity conditions (below-normal, normal, and high) represent inter-individual variability in blood rheology.**

| **Stenosis(%)** | **Low-viscosity**  **OSI（Pa）** | **Normal-viscosity**  **OSI(Pa)** | **High-viscosity**  **OSI(Pa)** |
| --- | --- | --- | --- |
| **30** | 0.3819 | 0.3815 | 0.4281 |
| **50** | 0.4554 | 0.4476 | 0.4260 |
| **70** | 0.4577 | 0.3904 | 0.4566 |
| **90** | 0.4427 | 0.4803 | 0.4639 |
